# Identification of Gut Microbiome Signatures Associated with Serotonin Pathway in Tryptophan Metabolism of Patients Undergoing Hemodialysis

**DOI:** 10.3390/ijms262110463

**Published:** 2025-10-28

**Authors:** Tien-Hsiang Kuo, Ping-Hsun Wu, Po-Yu Liu, Yun-Shiuan Chuang, Chi-Jung Tai, Mei-Chuan Kuo, Yi-Wen Chiu, Yi-Ting Lin

**Affiliations:** 1 School of Medicine, Kaohsiung Medical University, Kaohsiung 80708, Taiwan; thomas97747@gmail.com; 2 Division of Nephrology, Department of Internal Medicine, Kaohsiung Medical University Hospital, Kaohsiung Medical University, Kaohsiung 80708, Taiwan; 970392@kmuh.org.tw (P.-H.W.); mechku@kmu.edu.tw (M.-C.K.); chiuyiwen@kmu.edu.tw (Y.-W.C.); 3 Center for Big Data Research, Kaohsiung Medical University, Kaohsiung 80708, Taiwan; 4 Research Center for Precision Environmental Medicine, Kaohsiung Medical University, Kaohsiung 80708, Taiwan; 5 Faculty of Medicine, College of Medicine, Kaohsiung Medical University, Kaohsiung 80708, Taiwan; 6 Biomedical Artificial Intelligence Academy, Kaohsiung Medical University, Kaohsiung 80708, Taiwan; 7 School of Medicine, College of Medicine, National Sun Yat-sen University, Kaohsiung 80424, Taiwan; 8 Department of Family Medicine, Kaohsiung Medical University Hospital, Kaohsiung Medical University, Kaohsiung 80708, Taiwan; kinkipag@gmail.com (Y.-S.C.); taichijung@gmail.com (C.-J.T.); 9 Department of Family Medicine, School of Medicine, College of Medicine, Kaohsiung Medical University, Kaohsiung 80708, Taiwan; 10 Department of Family Medicine, Pingtung Hospital, Ministry of Health and Welfare, Pingtung 90003, Taiwan

**Keywords:** gut microbiota, tryptophan metabolism, chronic kidney disease, end-stage renal disease, hemodialysis

## Abstract

Serotonin, a tryptophan metabolite, exerts a significant influence on both brain and gut functionality. While previous research has elucidated the intricate dynamics of the gut–brain axis, the interplay between serotonin pathway metabolites and gut microbiota in individuals undergoing hemodialysis remains largely unexplored. Therefore, this study aimed to investigate gut microbiota composition corresponding to serotonin pathway metabolite levels among patients with hemodialysis. A total of 85 patients undergoing hemodialysis were selected. Their gut microbiota was analyzed using shotgun metagenomic sequencing profiling. The serotonin pathway metabolites, including 5-hydroxytryptophan (5-HTP), serotonin, 5-methoxytryptophan (5-MTP), 5-methoxytryptamine, melatonin, and 6-hydroxymelatonin, were analyzed with the liquid chromatograph–tandem mass spectrometer. The robust linear discriminant analysis Effect Size (LEfSe) was employed to reveal the gut microbiota signature according to levels of serotonin pathway metabolites. A significant β-diversity difference in 5-Methoxytryptamine (*p* = 0.037) was found, while no variance in α-diversity was detected. Using LefSe analysis, we identified an enriched Tannerellaceae family in the high-hydroxytryptophan (5-HTP) group, the Odoribacteraceae family in the high-serotonin group, the Eubacteriales order in the high-5-methoxytryptophan (5-MTP) group, the *Prevotella copri* species in the high-5-Methoxytryptamine group, and the *Clostridium* genus in the high-melatonin group. In contrast, an enriched *Clostridiaceae* family in the low-5-HTP group, the *Clostridiaceae* family in the low-serotonin group, and the *Bacteroides ovatus* species in the low-5-MTP group were found. Distinct gut microbiota signatures linked to serotonin pathway metabolites were identified in patients undergoing hemodialysis. These findings provide insights for future gut–brain axis research and may guide methods to modulate gut microbiota to influence serotonin metabolites.

## 1. Introduction

Patients undergoing hemodialysis presented significant differences in the composition of the gut microbiota compared to healthy controls, a condition commonly referred to as gut microbiota dysbiosis [1,2,3,4]. The gut microbiome, predominated by *Bacteroidetes* and *Firmicutes*, comprises trillions of microorganisms forming a complicated ecosystem engaged in host metabolism, nutrition, physiology, and immune functions [3,5,6,7]. Hemodialysis can induce structural and functional alterations in the gut microbiota, potentially exacerbating pre-existing clinical conditions [3,4,8]. Furthermore, gut microbiota dysbiosis may also contribute to adverse complications in hemodialysis [3,4,9]. Gut microbiota dysbiosis is characterized by several key features, including reduced microbial diversity, compromised barrier integrity, and the proliferation of harmful microorganisms, that are linked to pathological conditions [10,11]. Reduced microbial α diversity, resulting from dysbiosis, has been associated with higher mortality rates among hemodialysis patients, underscoring the crucial role of diversity in maintaining a healthy microbiome [12]. The *Proteobacteria*, *Actinobacteria*, and *Firmicutes* were found to increase in hemodialysis patients compared to the healthy controls [13].

Furthermore, gut microbial dysbiosis can significantly impact various physiological and metabolic pathways by producing specific classes of metabolites, including bile acids, short-chain fatty acids, branched-chain amino acids, trimethylamine N-oxide, tryptophan, and indole derivatives [14,15]. The tryptophan metabolism encompasses three distinct branches: the kynurenine pathway, the serotonin pathway, and the indole pathway [3,16]. In the serotonin pathway, gut microbiota can metabolize tryptophan as a precursor for the synthesis of serotonin, melatonin, and other metabolites [17].

Serotonin pathway metabolites encompass 5-hydroxytryptophan (5-HTP), serotonin (5-HT), 5-methoxytryptophan (5-MTP), melatonin, and 6-hydroxymelatonin. These compounds have been demonstrated to exert physiological effects through various signaling cascades. Serotonin’s primary function is to act via 5-HT receptors, thereby regulating gut motility, mood, and secretion [18,19], while melatonin signals through MT1 and MT2 receptors to affect circadian rhythm, oxidative stress, and immune modulation [20,21]. 5-MTP and melatonin also suppress inflammation via the MAPK and NF-κB pathways [22,23]. It is evident that gut microbes have the capacity to modulate the production of these metabolites. In turn, these metabolites may influence microbial diversity and function, thus forming a bidirectional communication loop through the gut–brain axis [24,25].

While previous research has made substantial progress in elucidating the connections between tryptophan metabolism and the gut–brain axis [16,17], the interplay between serotonin pathway metabolites and gut microbiota in individuals undergoing hemodialysis remains largely unexplored. Therefore, the primary objective of this study is to investigate associations between gut microbiota composition and peripheral serotonin (5-HT) levels in hemodialysis patients, given the physiological importance of gut-derived serotonin. Secondary exploratory analyses were performed for related serotonin pathway metabolites (5-HTP, melatonin, 5-MTP, and 6-hydroxymelatonin) to generate hypotheses for future studies.

## 2. Results

### 2.1. Baseline Characteristics

The baseline characteristics are summarized among six serotonin pathway-associated metabolites in Table 1. A total of 85 participants were included, and they were separated into high-level and low-level groups using median-based group stratification. The sex, presence of diabetes mellitus (DM), hypertension (HTN), coronary artery disease (CAD), albumin, urea clearance index (Kt/V (D)), and high-sensitivity C-reactive protein (hs-CRP) levels were similar between the high and low groups for all metabolites. Proton pump inhibitor (PPI) use was significantly different between the high and low groups for 5-HTP (*p* = 0.014); hemodialysis vintage showed a significant difference for 5-HTP (*p* = 0.014) and serotonin (*p* = 0.041); and the mean age was significantly different for 5-Methoxytryptamine (*p* = 0.010), as shown in Table 1.

### 2.2. Differential Abundance and Diversity of Gut Microbiota in Relation to Serotonin-Associated Metabolite Concentrations

**Figure 1:**
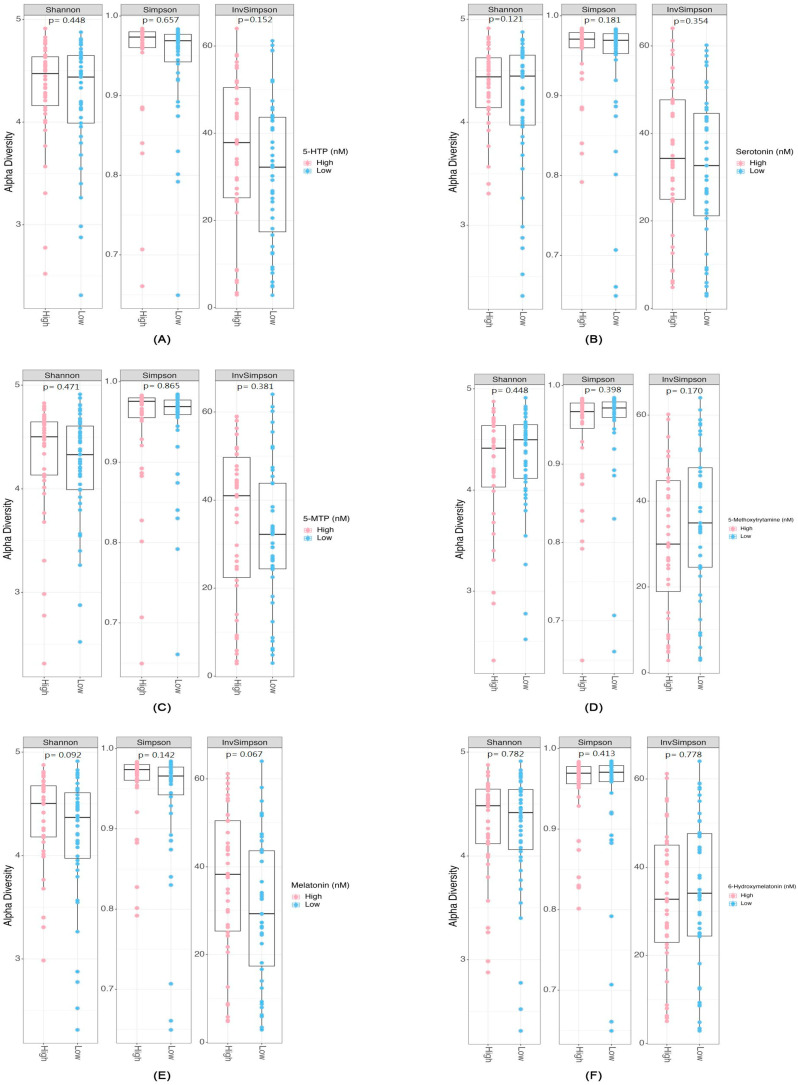
Alpha Diversity Corresponding to Different Levels of Serotonin Metabolites from the Tryptophan Pathway: (**A**) 5-HTP, (**B**) Serotonin, (**C**) 5-MTP, (**D**) 5-Methoxytryptamine, (**E**) Melatonin, and (**F**) 6-Hydroxymelatonin.

**Figure 2:**
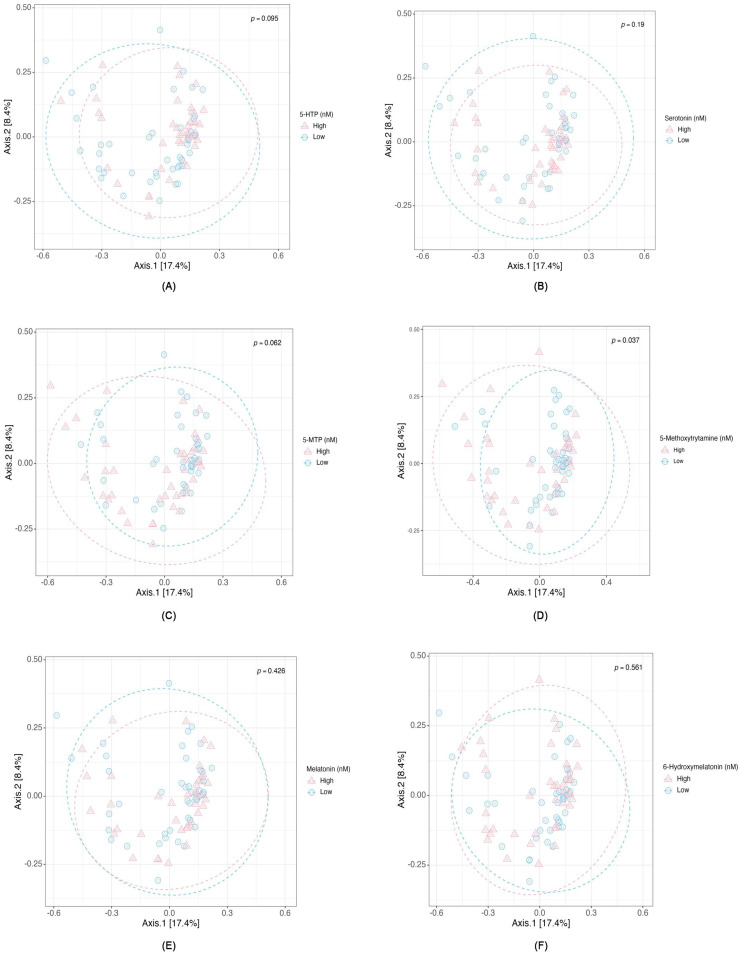
Beta Diversity Corresponding to Different Levels of Serotonin Metabolites from the Tryptophan Pathway: (**A**) 5-HTP, (**B**) Serotonin, (**C**) 5-MTP, (**D**) 5-Methoxytryptamine, (**E**) Melatonin, and (**F**) 6-Hydroxymelatonin.

We illustrate the differential abundance of genera and species in response to levels of metabolites along the serotonin pathway. Supplementary Figures S1–S6 illustrate the relative abundances of genera and species in relation to each serotonin pathway metabolite, highlighting the consistent genus-level predominance of *Bacteroides* and species-level predominance of *Prevotella* the unique microbial distribution influenced by distinct metabolites, specifically 5-HTP (Supplementary Figure S1), serotonin (Supplementary Figure S2), 5-MTP (Supplementary Figure S3), 5-Methoxytryptamine metabolite (Supplementary Figure S4), melatonin (Supplementary Figure S5), and 6-Hydroxymelatonin (Supplementary Figure S6). At the genus level, *Bacteroides* was consistently the most abundant taxon across all metabolite groups, reflecting its overall dominance in the gut microbiome. However, when examined at the species level, certain *Prevotella* species emerged as the most abundant, which explains the apparent difference between genus- and species-level profiles (Supplementary Figures S1–S6). The computation of α diversity indices, which quantifies the microbial richness and evenness within each patient group, showed that no significant difference was found in high and low concentrations of six serotonin pathway-associated metabolites in Shannon, Simpson, or inverse Simpson indices (Figure 1A–F). These α-diversity results were included to ensure a comprehensive evaluation of microbial diversity, despite the absence of significant differences. Regarding beta diversity, which captures inter-group dissimilarities, PCoA showed significant compositional differences only in high and low levels of 5-methoxytryptamine (*p* = 0.037) (Figure 2), while the other five metabolites demonstrated no significant difference in beta diversity.

### 2.3. Associations Between Serotonin Concentration, Microbial Composition, and Metabolic Pathways

The relationship between serotonin and microbial composition is shown in Supplementary Figure S7 by using Linear discriminant analysis Effect Size (LEfSe). Among those significantly associated species, a further comparison of the normalized (z-score standardized) relative abundance between high and low levels of serotonin was shown in Supplementary Figure S11. Following these observations, we determined, as detailed in Table 2, that specific species, including *Bacteroides stercoris* CAG:120, *Bacteroides* sp. AM16-15 and *Helicobacter felis* were associated with the up-regulation of serotonin. Conversely, *Rhizobium* sp. ASV8 and *Eubacterium* sp. CAG:180 was linked with its down-regulation. Among these identified species, only *Helicobacter felis* was significant under multiple testing correction, based on adjusted *p*-values. Additionally, Supplementary Figure S12 showed the distinct metabolic modules responding to varying serotonin concentrations. High serotonin levels characterize an enrichment in the trehalose degradation module. Contrarily, lower concentrations of serotonin were associated with metabolic modules including allose degradation, pyruvate dehydrogenase complex, glutamate degradation I, and glycolysis (preparatory phase), showcasing the functional versatility of the gut microbiota in response to changes in serotonin levels.

### 2.4. Secondary Exploratory Analyses for Additional Serotonin Pathway Metabolites

Differentially abundant microbial taxa between high- and low-5-HTP concentration groups were identified using LEfSe, as shown in Supplementary Figure S8. Among those significantly associated species, a further comparison of the standardized relative abundance between high and low levels of 5-HTP is shown in Supplementary Figure S9. Following these observations, as detailed in Table 2, we found that several species, including *Bacteroides xylanisolvens*, *Anaerotignum lactatifermentans*, *Streptococcus parasanguinis*, and *Bacteroides finegoldii* CAG:203, were associated with the up-regulation of 5-HTP. In contrast, *Roseburia hominis and Sutterella* sp. were associated with the down-regulation of 5-HTP. Among these identified species, only *Streptococcus parasanguinis* was significant in multiple tests according to adjusted *p*-values. Furthermore, Supplementary Figure S10 demonstrated the distinctive metabolic pathways responding to varying 5-HTP concentrations. When 5-HTP levels were high, we observed an enrichment in the lactate consumption II module. Conversely, lower 5-HTP levels were characterized by the prevalence of the cysteine degradation I, pyruvate: ferredoxin oxidoreductase, pentose phosphate pathway (non-oxidative branch), and threonine degradation II modules. These results illustrate the adaptive function of the gut microbiota responding to changes in 5-HTP levels.

The relationship between serotonin and microbial composition is shown in Supplementary Figure S7 by using LEfSe. Among those significantly associated species, a further comparison of the standardized relative abundance between high and low levels of serotonin is shown in Supplementary Figure S11. Following these observations, we determined, as detailed in Table 2, that specific species, including *Bacteroides stercoris* CAG:120, *Bacteroides* sp. AM16-15 and Helicobacter felis are associated with up-regulation of serotonin. Conversely, *Rhizobium* sp. ASV8 and *Eubacterium* sp. CAG:180 was linked with its down-regulation. Among these identified species, only *Helicobacter felis* was significant under multiple testing correction, based on adjusted *p*-values. Additionally, Supplementary Figure S12 shows the distinct metabolic modules responding to varying serotonin concentrations. High serotonin levels characterize an enrichment in the trehalose degradation module. Contrarily, lower concentrations of serotonin were associated with metabolic modules including allose degradation, pyruvate dehydrogenase complex, glutamate degradation I, and glycolysis (preparatory phase), showcasing the functional versatility of the gut microbiota in response to changes in serotonin levels.

The relationship between 5-MTP and gut microbial composition is shown in Supplementary Figure S13 by using LEfSe. Among those significantly associated species, a further comparison of the standardized relative abundance between high and low levels of 5-MTP is shown in Supplementary Figure S14. This exploration leads us to the critical findings in Table 2, where we identify that *Oscillibacter* sp. and *Ruminococcus* sp. are associated with the up-regulation of 5-MTP. Conversely, *Bacteroides ovatus* was associated with the down-regulation of 5-MTP. However, these identified species were not significant in multiple testing due to the limited sample sizes. Delving further into the functional aspect, Supplementary Figure S15 uncovers distinct gut metabolic modules corresponding to the varying 5-MTP concentrations. Low levels of 5-MTP were associated with the enrichment of ethanol production II, nitrate reduction (dissimilatory), and proline degradation. Interestingly, no metabolic module was found in the high concentration of 5-MTP. These results illustrate the adaptive function of the gut microbiota responding to changes in 5-MTP levels.

The relationship between 5-Methoxytryptamine and gut microbiota composition is shown in Supplementary Figure S16 by using LEfSe. Among those significantly associated species, a further comparison of the standardized relative abundance between high and low levels of 5-methoxytryptamine is shown in Supplementary Figure S17. *Prevotella lascolaii* and *Clostridium* sp. AM33-3 was associated with the up-regulation of 5-Methoxytryptamine, as indicated in Table 2. In contrast, *Parabacteroides johnsonii* CAG:246 was associated with the down-regulation of 5-Methoxytryptamine. However, these identified species were not significant in multiple testing due to the limited sample sizes. Supplementary Figure S18 demonstrates the differential metabolic pathways reacting to varying concentrations of 5-methoxytryptamine. An increase in 5-Methoxytryptamine concentration was associated with the enhancement of specific metabolic modules, including pentose phosphate pathway (non-oxidative branch), glutamine degradation II, glycolysis (pay-off phase), fructan degradation, and threonine degradation II. However, no metabolic module was found in the low concentration of 5-methoxytryptamine. This presents a comprehensive picture of how microbial composition and metabolic activity changes align with 5-methoxytryptamine concentration variations.

The relationship between melatonin and gut microbiota composition is shown in Supplementary Figure S19 by using LEfSe. Among those significantly associated species, a further comparison of the standardized relative abundance between high and low levels of melatonin is shown in Supplementary Figure S20. Based on these observations, we determined, as detailed in Table 2, that specific species, including Clostridium sp., AT4, *Phascolarctobacterium faecium*, and *Clostridium* sp. 27_14, were associated with the up-regulation of melatonin. No species was found to be linked to its down-regulation. Additionally, Supplementary Figure S21 showed the distinct metabolic modules responding to varying melatonin concentrations. High melatonin levels were characterized by an enrichment in the glycerol degradation II module. In contrast, lower Melatonin concentrations were associated with metabolic modules, such as the triacylglycerol degradation module, highlighting the gut microbiota’s functional versatility in response to changes in Melatonin levels.

The relationship between 6-Hydroxymelatonin and gut microbiota composition is shown in Supplementary Figure S22 by using LEfSe. Among those significantly associated species, a further comparison of the standardized relative abundance between high and low levels of 6-Hydroxymelatonin is shown in Supplementary Figure S23. This exploration leads us to the critical findings in Table 2, where we identify that *Bifidobacterium longum* and *Clostridium symbiosum* were associated with the up-regulation of 6-Hydroxymelatonin. Conversely, *Bacteroides neonati* and *Bacteroides* sp. CAG:875 were associated with the down-regulation of 6-Hydroxymelatonin. However, these identified species were not significant in multiple testing due to the limited sample sizes. Delving further into the functional aspect, Supplementary Figure S24 uncovers distinct gut metabolic modules corresponding to the varying 6-Hydroxymelatonin concentrations. High levels of 6-Hydroxymelatonin were associated with the enrichment in the glycerol degradation I module. In contrast, low 6-Hydroxymelatonin levels were associated with the tryptophan degradation module. These results illustrate the adaptive function of the gut microbiota in response to changes in 6-Hydroxymelatonin levels.

## 3. Discussion

Our findings reveal a nuanced interplay between serotonin pathway metabolites and gut microbiota in hemodialysis patients, suggesting that these metabolic shifts may reflect or contribute to alterations in the microbial ecosystem in this population. For instance, the identification of *Streptococcus parasanguinis* in association with elevated 5-HTP levels raises questions about its potential involvement in modulating serotonin precursor availability or influencing gut neuroendocrine signaling. Similarly, *Helicobacter felis*, enriched in the high-serotonin group, might be implicated in the microbial synthesis or regulation of serotonin, given its known expression of tryptophan hydroxylase. The presence of *Clostridium* species in relation to high-melatonin levels may also point to species-specific metabolic roles in melatonin biosynthesis or degradation. Conversely, the reduction in Bacteroides *ovatus* in the low-5-MTP group suggests a possible link between microbial dysbiosis and impaired production of anti-inflammatory metabolites. Moreover, the distinct enrichment patterns in microbial metabolic modules underscore the adaptability of the gut microbiota to varying environments of serotonin metabolites, reflecting potential compensatory mechanisms or altered substrate availability in the context of renal dysfunction. These microbiota–metabolite associations contribute to our understanding of gut–brain–liver axis dynamics and may offer novel insights for therapeutic modulation of the gut ecosystem in patients with HD.

### 3.1. Microbial Diversity and Serotonin Pathway-Associated Metabolites

Microbial diversity showed no significant differences in α-diversity indices (Shannon, Simpson, and InvSimpson) across six serotonin pathway metabolites. A modest but statistically significant beta diversity difference was found in 5-methoxytryptamine groups, though melatonin groups showed no significant differences, contrary to the findings of Iesanu et al. [20]. We acknowledge that the observed beta-diversity signal for 5-methoxytryptamine is weak and should be interpreted cautiously, as it may reflect subtle shifts in microbial composition rather than broad community restructuring. The relatively small sample size (N = 85) in this study may have limited our statistical power to detect subtle differences in alpha diversity. Larger sample sizes typically enhance the ability to detect smaller but biologically relevant variations in microbial diversity, particularly when evaluating conditions such as hemodialysis, which inherently exhibit significant microbiome heterogeneity. An important methodological consideration is that our study did not include a healthy control group or baseline (pre-hemodialysis) fecal samples from the enrolled patients. This design precludes direct conclusions about whether hemodialysis per se drives the observed alterations, as potential confounding by chronic kidney disease and long-term dialysis exposure cannot be ruled out. These limitations should be kept in mind when interpreting our findings.

### 3.2. Species Associated with Metabolites Involved in Serotonin Pathway

5-hydroxytryptophan (5-HTP), a serotonin precursor that crosses the blood–brain barrier, influences various physiological effects [26,27,28]. Higher abundances of *Bacteroides* species (*xylanisolvens*, *reticulotermitis*, *finegoldii*, and *propionicifaciens*) were found in the 5-HTP-treated groups [26], though some *Bacteroides* species with TnaA enzyme may convert 5-HTP to 5-hydroxyindole [27]. Regarding serotonin relationships, studies showed increased *Sutterella* genus in Alzheimer’s disease rats with reduced brain serotonin [29]. Helicobacter pylori expressed tryptophan hydroxylase [30], and our study showed an enriched Helicobacteraceae family in high-serotonin groups (Supplementary Figure S11). While *Clostridium ramosum* up-regulates peripheral 5-HT [31], our study found higher *Clostridiaceae* abundance in low-serotonin groups. *Proteobacteria* showed a positive association with serotonin levels [32], consistent with our findings of higher *levels of Desulfovibrio desulfuricans and Delftia* sp. ASV31 and *Helicobacter felis* abundance. *Bacteroides fragilis* showed higher abundance in high-serotonin groups, contradicting Yano et al.’s findings [24]. 5-Methoxytryptophan (5-MTP) demonstrates protective biological effects [22,33,34]. *Oscillibacter* sp. enrichment in high-5-MTP groups aligned with previous mouse model studies [35]. Our findings identified correlations with *Oscillibacter* sp., *Ruminococcus* sp., and *Bacteroides ovatus*. 5-Methoxytryptamine (5-MT) reduces body weight and improves glucose tolerance [23], with significant correlations to gut microbiome composition in post-stress disordered rats [36]. 5-methoxytryptamine (5-MT), a derivative of serotonin, has been reported to influence energy metabolism and anti-inflammatory pathways distinctly from other metabolites of the serotonin pathway. Specifically, 5-MT modulates macrophage polarization, influencing inflammation and insulin sensitivity, potentially creating a unique gut environment that selectively enriches specific microbial communities. This distinct immunometabolic role might explain the unique microbial beta-diversity patterns associated with 5-MT compared to those of other metabolites. Our study identified associations with *Prevotella copri*, *Mailhella massiliensis*, *Prevotella lascolaii*, and *Parabacteroides johnsonii* CAG:246. Melatonin influences various body functions [20,37]. Studies showed reduced *Firmicutes*/*Bacteroidetes* ratios [25] and restored *Bacteroidaceae* abundances after melatonin administration [38]. While previous research found reduced *Clostridium perfringens* abundance with melatonin treatment [39], our study showed higher Clostridium sp. abundance. AT4 and *Clostridium* sp. 27_14 in high-melatonin groups (Supplementary Figure S20). 6-hydroxymelatonin, a melatonin metabolite formed in the liver [40], demonstrates free radical scavenging properties [41] and acts as an MT1 receptor agonist and an MT2 receptor partial agonist [21]. Our study identified associations with *Flavonifractor plautii*, *Bifidobacterium longum*, *Muribaculaceae bacterium*, *Clostridium symbiosum*, and *Bacteroides neonati*. The metabolic modules identified, including cysteine degradation and pyruvate: ferredoxin oxidoreductase, could mechanistically impact tryptophan and 5-HTP metabolism by modulating precursor availability or through indirect pathways such as oxidative stress management and energy balance. These biochemical shifts in gut metabolism might influence serotonin synthesis, availability, and systemic distribution, impacting both gut and brain functions through gut–brain communication pathways. Interestingly, we observed statistically significant, opposing associations between hemodialysis vintage and the levels of serotonin and its precursor, 5-hydroxytryptophan (5-HTP). Specifically, longer dialysis vintage correlated with higher 5-HTP levels but lower serotonin levels. This seemingly paradoxical finding might reflect progressive alterations in gut microbiota composition and functionality with prolonged dialysis exposure, potentially influencing the efficiency of serotonin synthesis or degradation pathways. Chronic hemodialysis is known to progressively impact gut microbiota through repeated metabolic and osmotic stresses, changes in gut barrier integrity, and recurrent inflammatory episodes, each of which may selectively affect microbial populations involved in serotonin metabolism. Such dialysis-associated microbiome shifts could decrease the conversion efficiency from 5-HTP to serotonin or increase microbial or host-mediated serotonin catabolism over time, resulting in the observed inverse relationship. This finding highlights the importance of considering dialysis vintage as a crucial modifier when interpreting microbial and metabolite associations in hemodialysis populations, and underscores the necessity of longitudinal studies to dissect temporal relationships and underlying mechanisms further.

### 3.3. Gut Metabolic Modules and Serotonin Pathway Metabolites Relationship

Regarding gut metabolic modules, pyruvate: ferredoxin oxidoreductase and threonine degradation II modules were enriched in the low-5-HTP groups [24,42,43]. The pyruvate dehydrogenase complex module was enriched in the low-serotonin groups, possibly due to microbiota-producing tryptophanase converting tryptophan to indole and pyruvate [3,4,44]. Higher 5-methoxytryptamine levels showed enriched glutamine degradation II modules, consistent with glutamine’s role in up-regulating tryptophan hydroxylase levels [45]. Low melatonin levels correlated with increased activity of triacylglycerol degradation pathways, suggesting that gut microorganisms influence melatonin synthesis [20,46]. For example, the enrichment of the pyruvate dehydrogenase complex and glutamate degradation modules in the low-serotonin groups may reflect microbial shifts toward energy harvesting and amino acid utilization under conditions of reduced serotonin availability. These functional adaptations potentially influence serotonin biosynthesis indirectly by altering tryptophan availability or directly through competitive substrate utilization. Such metabolic shifts could subsequently affect clinical outcomes in hemodialysis patients, including altered inflammatory states and nutritional status, warranting further exploration. Microbial taxa identified in our study, such as *Helicobacter felis* and *Clostridium* species, possess enzymatic machinery (e.g., tryptophan hydroxylase) that can directly influence serotonin biosynthesis. The identified metabolic modules, such as glutamine degradation and pyruvate metabolism, may similarly provide substrates or modulate gut environments that facilitate or suppress microbial serotonin synthesis pathways, thus highlighting complex host–microbe interactions relevant to serotonin metabolism. The identified gut metabolic modules, such as glutamine and triacylglycerol degradation, may influence metabolite availability, particularly amino acids essential for neurotransmitter synthesis and signaling molecules crucial for gut–brain communication. These metabolic adjustments potentially modulate host physiology directly through altered serotonin production or indirectly through metabolic intermediates influencing neural signaling pathways. These observed microbial and metabolic shifts might significantly influence serotonin signaling along the gut–brain axis, potentially affecting cognitive functions, mood regulation, and gastrointestinal symptoms commonly observed in hemodialysis patients. Understanding these connections could be pivotal for developing microbiota-based therapeutic strategies aimed at improving neurological and psychological outcomes in this patient population.

### 3.4. Study Limitations

This study has several limitations for the following reasons. First, this is a cross-sectional study that could only assess relative abundance and analyses correlations between microbes and metabolites at a single time point. Second, 16s rRNA was limited because it could only be analyzed at the genus level. Third, participants were divided into “high” and “low” metabolite groups based on median values, which are not established clinical thresholds and may limit the generalizability of results. Although we adjusted for key demographic factors such as age and sex in subsequent regression analyses, residual confounding might still exist, potentially impacting the reliability and interpretability of our results. Fourth, the lack of a healthy or CKD-stage-matched control group prevents definitive conclusions about disease-specific microbial alterations and limits the interpretability of metabolite-level stratification based solely on within-cohort distributions. Fifth, the small sample size and unmeasured confounders, such as dialysis vintage, diet, and comorbidities, may influence the observed associations. Sixth, although we focused on serotonin pathway metabolites, key intermediates such as tryptophan and indole derivatives were not measured, which limits mechanistic insight into microbiota-mediated serotonin metabolism. Finally, relevant clinical symptoms or biomarkers potentially associated with serotonin, such as gastrointestinal motility or mood disorders, were not systematically recorded, limiting translational interpretation. Furthermore, our analysis focused primarily on LEfSe for the exploratory identification of microbial taxa associated with serotonin metabolites, and was validated using linear regression models. Additional approaches such as canonical correlation analysis (CCA), redundancy analysis (RDA), or correlation-based analyses (Spearman or Pearson) could provide complementary insights, especially for exploring complex multi-dimensional microbiome–metabolite relationships. Future studies with larger sample sizes should consider incorporating these methods to comprehensively validate and extend our findings.

## 4. Materials and Methods

### 4.1. Study Population

The study protocols were approved by the Ethics Committee of Kaohsiung Medical University Hospital (KMUHIRB-E(I)-20160095 and KMUHIRB-E(I)-20180118). All participants provided written informed consent. Hemodialysis patients were recruited from the dialysis unit at Kaohsiung Medical University Hospital, Taiwan, from August 2017 to February 2018, as part of the Taiwan Kidney Outcome Omics study (TAKOO). Eligible participants were those who received regular hemodialysis three times per week, for 3.5–4 h with high-flux dialyzers. Participants with active malignancies or participants who had been prescribed antibiotics within three months before enrollment were excluded. Fecal samples were collected from 85 stable HD patients and analyzed by high-throughput shotgun metagenomics sequencing. From this registry, it was possible to extract individual-level information on factors such as age, sex, medical history (use of PPIs or not), and biochemical data (albumin and hs-CRP) for all participants.

### 4.2. Comorbidities and Laboratory and Clinical Variables

Diabetes mellitus (DM) was defined as patients with HbA1C of 6.5% or higher or taking antidiabetic drugs. Hypertension was identified in those with a blood pressure of 140/90 mmHg or higher, or who were taking oral antihypertensive medicines. Coronary artery disease (CAD) was defined as a history of angina and ischemic electrocardiogram change, old myocardial infarction, or having undergone coronary bypass surgery or angioplasty. Blood samples were routinely collected from the arteriovenous fistula or graft in the morning from fasting subjects in the second hemodialysis session. Biochemical data, including albumin and hsCRP, were collected from routine data within 30 days of the stool sample collection and analyzed in the hospital central lab. Albumin and hs-CRP were measured by electro-chemiluminescent immunoassay (ECLIA) (Cobas e601, Roche Diagnostics, Germany).

### 4.3. Serotonin Pathway-Associated Metabolite Measurement

The serotonin-related metabolites (5-hydroxytryptophan [5-HTP], serotonin, 5-methoxytryptophan [5-MTP], 5-methoxytryptamine, melatonin, and 6-hydroxymelatonin) were measured in this study. The serum samples were mixed with ice-cold acetonitrile containing the internal standard to precipitate proteins. After vortexing and centrifuge, the supernatants were transferred to fresh vials. Targeted metabolites were measured using ultra-performance liquid chromatography (UPLC) coupled with a XevoTM triple quadrupole mass spectrometer (Waters Corporation, Milford, MA, USA). In brief, an Acquity UPLC system with a 1.7-μm (2.1 × 100 mm) BEH C18 column (1.7 μm, 2.1 × 100 mm) was used to perform liquid chromatography with linear gradient conditions: 0–1.5 min, 1% B; 1.5–2.5 min, 5% B; 2.5–4.5 min, 100% B; 4.5–5.0 min, 100% B; 9.5–12 min, 99% A [solvent system A, water/formic acid (100:0.1, *v*/*v*); B, acetonitrile/formic acid (100:0.1, *v*/*v*)]. The injection volume was 2 μL, and the column temperature was maintained at 35 °C. Mass spectrometric detection was performed on a Xevo triple quadrupole mass spectrometer equipped with an electrospray ionization source operating in positive or negative ionization mode. The source parameters were capillary voltage, 3.0 kV; cone voltage, 30 V; source temperature, 150 °C; desolvation temperature, 600 °C; cone gas flow, 50 L/hr; and desolvation gas flow, 800 L/hr. The online mass spectrometry analysis used multiple reaction monitoring modes. Waters MarkerLynx XS software version 4.1 (Waters Corporation, Milford, MA, USA) was used for peak detection, alignment, and deconvolution of the UPLC-MS data. Metabolites were identified by matching their accurate masses, retention times, isotope patterns, and MS/MS fragmentation spectra against an in-house library of authenticated standards. Quantification was performed by generating calibration curves using standard solutions of 5-hydroxytryptophan, serotonin, 5-methoxytryptophan, 5-methoxytryptamine, melatonin, and 6-hydroxymelatonin.

### 4.4. Microbiome Analysis

All stool samples were frozen immediately after collection by each participant and then delivered to the laboratory (Germark Biotechnology, Taichung, Taiwan) in cooler bags with ice packs to maintain a stable, low temperature within 24 h. Bacterial DNA was extracted from stool using the QIAamp Fast DNA Stool Mini Kit (Qiagen, Germantown, MD, USA). The DNA quantity and quality were determined using the NanoDrop ND-1000 (Thermo Scientific, Wilmington, DE, USA) with an acceptable OD 260/280 ratio of 1.8 to 2.0 to ensure high-quality DNA. Agarose gel electrophoresis was also employed to verify DNA integrity. The extracted DNA was stored at −80 °C until library construction and sequencing, ensuring quality preservation. Sequencing was conducted on an Illumina NovaSeq 6000 platform (San Diego, CA, USA), generating paired-end reads of 150 bp. Raw reads underwent quality control using the Kneaddata (version 0.7.433) pipeline, which incorporated Trimmomatic (version 0.39) for adapter trimming and low-quality base removal, Bowtie2 (version 2.4.2) for host read depletion (human hg38), and Komplexity (version 0.3.6) for low-complexity filtering. Metagenome assembly was performed using MEGAHIT (version 1.0) [47] to assemble quality-filtered reads into contigs. Taxonomic profiling was conducted through both read-based (Sourmash (version 2.0) [48] with GTDB database [49]) and contig-based (MMSeqs2 “easy-taxonomy” pipeline) [50,51] approaches. Functional annotation was carried out using Prokka (version 1.14.5) for ORF prediction, and KO, EC, and MetaCyc pathway assignments were obtained using MMSeqs2 and MinPath (version 1.1). Gene abundance was normalized using TPM (transcripts per million), calculated by mapping reads to contigs and aggregating ORF-level expression. Metagenome-assembled genomes (MAGs) were reconstructed using MetaBAT (version 2.17) and evaluated for completeness using CheckM (version 1.1.6). MAGs were considered high-quality if they exhibited ≥90% completeness and ≤5% contamination; medium-quality MAGs were defined by ≥50% completeness and ≤10% contamination. MAGs not meeting these criteria were excluded from subsequent analyses. The detailed descriptions of sequencing, quality control, assembly, and annotation were in the Supplementary Methods.

### 4.5. Bioinformatics and Statistical Analysis

All statistical analyses were performed using R software (version 4.2.2; R Core Team 2022) unless otherwise specified. In the data analysis section, our objective was to investigate the correlations between serotonin pathway metabolite concentrations and gut microbiome features, including metagenomic species (MGS) and gut metabolic modules (GMMs). To classify patients into comparison groups, we used a median split for each metabolite, dividing participants into high- and low-concentration groups. Participants were stratified into high- and low-concentration groups based on the median metabolite concentration to facilitate exploratory analyses. This approach was selected because no established clinical or biologically validated cut-off points for serotonin pathway metabolites exist in hemodialysis patients. The median-based splits should thus be viewed as hypothesis-generating rather than definitive. The analysis process was described in the Supplementary method. For microbiota-metabolite associations, we supplemented LEfSe with linear regression models adjusted for age and sex, and applied false discovery rate (FDR) correction to control for multiple comparisons. Only associations with FDR-adjusted *p*-values < 0.05 were considered significant.

## 5. Conclusions

In this study, we identified distinct gut microbiota signatures associated with metabolites of the serotonin pathway in patients undergoing HD. Specific microbial taxa, such as *Streptococcus parasanguinis*, *Helicobacter felis*, and *Clostridium* species, were linked to differential levels of serotonin-related metabolites, suggesting possible microbe–host interactions along the serotonin biosynthetic and metabolic pathways. Although global microbial diversity remained largely unchanged, metabolite-specific compositional and functional differences were noted, particularly for 5-methoxytryptamine. These findings provide novel insights into the potential roles of gut microbiota in modulating peripheral serotonin metabolism in the context of chronic kidney disease. Given the cross-sectional design and lack of functional validation, further longitudinal and mechanistic studies are necessary to explore causality, assess clinical relevance, and ascertain whether microbiota-targeted interventions could beneficially influence serotonin-mediated pathways in patients with HD.

## Figures and Tables

**Table 1:** Demographic and Clinical Characteristics of Study Participants in the Serotonin Pathway Analysis with Corresponding Metabolite Data (N = 85).

Parameters/Groups N (%) or Mean (SD) or Median [Q1; Q3]	5-HTP (nM)		*p*	Serotonin (nM)		*p*	5-MTP (nM)		*p*
	High (N = 42)	Low (N = 43)		High (N = 42)	Low (N = 43)		High (N = 42)	Low (N = 43)	
Age	60.2 (10.3)	60.8 (11.2)	0.783	58.2 (10.1)	62.7 (11.0)	0.052	58.3 (10.7)	62.7 (10.5)	0.064
Female	20 (47.6%)	16 (37.2%)	0.452	17 (40.5%)	19 (44.2%)	0.899	16 (38.1%)	20 (46.5%)	0.572
DM	10 (23.8%)	18 (41.9%)	0.124	14 (33.3%)	14 (32.6%)	1.000	13 (31.0%)	15 (34.9%)	0.877
HTN	29 (69.0%)	38 (88.4%)	0.056	32 (76.2%)	35 (81.4%)	0.748	30 (71.4%)	37 (86.0%)	0.166
CAD	7 (16.7%)	11 (25.6%)	0.459	5 (11.9%)	13 (30.2%)	0.072	9 (21.4%)	9 (20.9%)	1.000
PPI use	11 (26.2%)	2 (4.65%)	0.014	9 (21.4%)	4 (9.30%)	0.211	7 (16.7%)	6 (14.0%)	0.963
Albumin	3.91 (0.36)	3.91 (0.35)	0.931	3.89 (0.35)	3.93 (0.36)	0.526	3.91 (0.33)	3.92 (0.38)	0.897
Kt/V (D)	1.56 (0.19)	1.51 (0.22)	0.246	1.54 (0.22)	1.53 (0.20)	0.949	1.51 (0.18)	1.56 (0.23)	0.231
hs-CRP	3.87 (4.47)	5.09 (6.93)	0.340	3.81 (4.54)	5.17 (6.92)	0.291	4.35 (4.96)	4.62 (6.66)	0.835
Hemodialysis Vintage	111 (74.4)	72.7 (65.5)	0.014	75.4 (60.3)	107 (79.8)	0.041	97.9 (71.7)	85.3 (73.1)	0.425
Metabolites (nM)	2.74 [2.04; 6.65]	0.03 [0.00; 0.45]	<0.001	13.5 [10.7; 21.2]	6.36 [2.87; 27.2]	<0.001	21.8 [18.4; 27.1]	12.2 [9.54; 14.3]	<0.001
**Parameters/Groups** **N (%) or** **Mean (SD) or** **Median [Q1; Q3]**	**5-Methoxytryptamine (nM)**		***p***	**Melatonin** **(nM)**		***p***	**6-Hydroxymelatonin (nM)**		***p***
	**High (N = 40)**	**Low (N = 45)**		**High (N = 42)**	**Low (N = 43)**		**High (N = 42)**	**Low (N = 43)**	
Age	57.3 (11.5)	63.4 (9.21)	0.010	59.5 (9.72)	61.5 (11.7)	0.413	61.3 (11.6)	59.7 (9.90)	0.506
Female	18 (45.0%)	18 (40.0%)	0.806	16 (38.1%)	20 (46.5%)	0.572	17 (40.5%)	19 (44.2%)	0.899
DM	14 (35.0%)	14 (31.1%)	0.881	12 (28.6%)	16 (37.2%)	0.538	15 (35.7%)	13 (30.2%)	0.759
HTN	33 (82.5%)	34 (75.6%)	0.606	31 (73.8%)	36 (83.7%)	0.394	32 (76.2%)	35 (81.4%)	0.748
CAD	8 (20.0%)	10 (22.2%)	1.000	8 (19.0%)	10 (23.3%)	0.834	7 (16.7%)	11 (25.6%)	0.459
PPI use	5 (12.5%)	8 (17.8%)	0.709	7 (16.7%)	6 (14.0%)	0.963	8 (19.0%)	5 (11.6%)	0.516
Albumin	3.91 (0.36)	3.92 (0.42)	0.710	3.95 (0.30)	3.87 (0.40)	0.263	3.89 (0.38)	3.93 (0.32)	0.544
Kt/V (D)	1.56 (0.19)	1.54 (0.21)	0.873	1.54 (0.21)	1.53 (0.21)	0.899	1.53 (0.21)	1.54 (0.21)	0.698
hs-CRP	3.87 (4.47)	4.36 (5.83)	0.825	4.70 (6.08)	4.29 (5.70)	0.754	4.54 (6.42)	4.45 (5.34)	0.944
Hemodialysis Vintage	111 (74.4)	84.0 (67.3)	0.314	94.9 (69.9)	88.3 (75.2)	0.675	97.8 (78.7)	85.4 (65.7)	0.433
Metabolites (nM)	2.74 [2.04; 6.65]	0.90 [0.69; 1.02]	<0.001	0.69 [0.60; 1.73]	0.12 [0.06; 0.27]	<0.001	6.15 [4.79; 13.0]	2.22 [1.65; 2.91]	<0.001

Note: SD: standard deviation; Q1: the first quartile; Q3: the third quartile; DM: diabetes mellitus; HTN: hypertension; CAD: coronary artery disease; PPI: proton pump inhibitor; hs-CRP: high-sensitivity C-reactive protein.

**Table 2:** The Associations Between Signature MGS and Metabolites of the Serotonin Pathway by Linear Regression Model (n = 85).

Metabolites/MGS	Estimate	95% CI	*p*-Values	Adjusted *p*-Values	*R* ^2^
5-HTP (nM)					0.346
Up-Regulate					
* Bacteroides xylanisolvens*	1.30	(0.27, 2.32)	0.014 *	0.069 ^•^	
* Anaerotignum lactatifermentans*	0.49	(0.00, 0.98)	0.048 *	0.160	
* Streptococcus parasanguinis*	0.48	(0.19, 0.78)	0.002 **	0.016 *	
* Bacteroides finegoldii* CAG:203	−0.62	(−1.32, 0.08)	0.080 ^•^	0.172	
Down-Regulate					
* Sutterella sp. KLE1602*	0.05	(−0.22, 0.33)	0.704	0.704	
* Roseburia faecis*	−0.19	(−0.60, 0.22)	0.349	0.436	
* Roseburia hominis*	−0.24	(−0.58, 0.10)	0.169	0.282	
* Ruminococcaceae bacterium AF10-16*	−0.10	(−0.49, 0.29)	0.602	0.669	
* Sutterella sp.*	−0.21	(−0.58, 0.16)	0.267	0.382	
Serotonin (nM)					0.445
Up-Regulate					
* Bacteroides xylanisolvens*	−5.47	(−38.85, 27.91)	0.745	0.767	
* Bacteroides finegoldii*	10.19	(−9.49, 29.87)	0.305	0.663	
* Bacteroides stercoris CAG:120*	11.21	(−6.27, 28.69)	0.205	0.663	
* Bacteroides neonati*	−7.62	(−28.87, 13.64)	0.477	0.735	
* Parabacteroides johnsonii CAG:246*	−6.77	(−20.80, 7.26)	0.339	0.663	
* Bacteroides sp. CAG:633*	4.96	(−14.65, 24.57)	0.616	0.767	
* Bacteroides sp. AM16-15*	5.10	(−3.49, 13.68)	0.241	0.663	
* Helicobacter felis*	24.82	(14.18, 35.50)	<0.001 ***	<0.001 ***	
* Bacteroides sp. HPS0048*	1.95	(−11.10, 15.00)	0.767	0.767	
* Bacteroides congonensis*	1.72	(−7.76, 11.20)	0.719	0.767	
* Bacteroides fragilis CAG:558*	4.98	(−10.28, 20.23)	0.517	0.735	
Down-Regulate					
* Clostridium symbiosum*	−4.53	(−19.14, 10.09)	0.539	0.735	
* Rhizobium sp. ASV8*	−11.46	(−24.17, 1.26)	0.077 ^•^	0.383	
* Eubacterium sp. CAG:180*	−12.90	(−23.50, −2.29)	0.018 *	0.134	
5-MTP (nM)					0.255
Up-Regulate					
* Oscillibacter sp.*	0.84	(−0.41, 2.09)	0.186	0.464	
* Roseburia intestinalis*	−0.42	(−1.77, 0.94)	0.543	0.991	
* Eubacterium rectale*	0.15	(−1.36, 1.66)	0.846	0.991	
* Barnesiella intestinihominis*	0.00	(−0.58, 0.58)	0.991	0.991	
* Ruminococcus bromii*	0.17	(−0.76, 1.10)	0.716	0.991	
* Ruminococcaceae bacterium TF06-43*	0.07	(−1.12, 1.25)	0.912	0.991	
* Ruminococcus sp.*	0.98	(−0.03, 1.98)	0.056 ^•^	0.187	
Down-Regulate					
* Bacteroides ovatus*	−2.91	(−5.13, −0.69)	0.011 *	0.054 ^•^	
* Ruminococcus gnavus*	−0.02	(−1.14, 1.11)	0.978	0.991	
5-Methoxytryptamine (nM)					0.102
Up-Regulate					
* Prevotella copri*	0.10	(−0.10, 0.29)	0.332	0.665	
* Mailhella massiliensis*	−0.04	(−0.26, 0.16)	0.668	0.842	
* Prevotella sp. CAG:279*	0.03	(−0.17, 0.24)	0.748	0.842	
* Prevotella lascolaii*	0.15	(−0.10, 0.41)	0.232	0.626	
* Clostridium sp. AM33-3*	0.14	(−0.07, 0.35)	0.184	0.626	
* Sutterella sp.*	0.02	(−0.18, 0.21)	0.842	0.842	
Down-Regulate					
* Parabacteroides johnsonii CAG:246*	−0.06	(−0.29, 0.18)	0.634	0.842	
Melatonin (nM)					0.195
Up-Regulate					
* Clostridium sp. AT4*	0.22	(0.03, 0.40)	0.021 *	0.052	
* Phascolarctobacterium faecium*	−0.28	(−0.46, −0.11)	0.002 **	0.010 *	
* Bacteroides sp. 519*	0.05	(−0.26, 0.37)	0.728	0.728	
* Clostridium sp. 27_14*	0.22	(−0.00, 0.45)	0.053 ^•^	0.088 ^•^	
6-Hydroxymelatonin (nM)					0.096
Up-Regulate					
* Flavonifractor plautii*	−0.74	(−4.10, 2.63)	0.664	0.885	
* Bifidobacterium longum*	0.58	(−0.43, 1.59)	0.255	0.885	
* Muribaculaceae bacterium*	0.58	(−1.150, 2.31)	0.507	0.885	
* Clostridium symbiosum*	1.43	(−0.47, 3.34)	0.139	0.885	
* Blautia sp. CAG:257*	0.96	(−1.00, 2.91)	0.333	0.885	
Down-Regulate					
* Bacteroides neonati*	−0.55	(−2.88, 1.78)	0.640	0.885	
* Bacteroides sp. CAG:875*	0.30	(−1.85, 2.44)	0.783	0.895	

Note: ^•^ *p* < 0.1, * *p* < 0.05, ** *p* < 0.01, *** *p* < 0.001; CI: confidence interval.

## Data Availability

Due to ethical restrictions imposed by the Institutional Review Board (IRB) of Kaohsiung Medical University Hospital, the raw metagenomic sequencing data are not publicly available. Researchers wishing to access the raw sequencing data for academic and scientific purposes must first obtain appropriate IRB approval at their respective institutions. After receiving IRB authorization, requests for data access can be directed to the corresponding author, who will facilitate data sharing in accordance with the relevant ethical guidelines.

## Supplementary Materials

The following supporting information can be downloaded at: https://www.mdpi.com/article/10.3390/ijms262110463/s1. Refs. [47,48,49,50,51,52,53,54,55,56,57,58,59,60,61,62,63,64,65,66] are cited in the Supplementary Materials.
