# Supplementary material for: Identification of Gut Microbiome Signatures Associated with Serotonin Pathway in Tryptophan Metabolism of Patients Undergoing Hemodialysis
Source: Int J Mol Sci. 2025 Oct 28;26(21):10463. doi: 10.3390/ijms262110463 (PMC12610804; doi:10.3390/ijms262110463)

Supplementary Material

Identification of gut microbiome signatures associated with serotonin pathway in tryptophan metabolism in patients undergoing hemodialysis

Supplementary Methods

Metagenomics sequencing and Raw reads quality control

Extracted DNA (about 500 ng) was fragmented to approximately 350 base pairs by the Covaris S2 system (Covaris, Inc., Woburn, MA, USA) and then subjected to library construction with the Illumina DNA Prep Kit (Illumina, San Diego, CA). Sequencing is performed using an Illumina NovaSeq 6000 sequencer, resulting in paired-end reads of 150 bp in length. On a per-sample basis, read quality control (QC) is performed using the Kneaddata (version 0.7.433) pipeline (https://github.com/biobakery/kneaddata), which integrates Trimmomatic (version 0.39) [1] for trimming Illumina adaptors and low-quality regions, filtering short reads and Bowtie2 (version 2.4.2) [2] for identifying and removing host contaminations from human hg38 build (or mouse mm10 build) and PhiX genome. Reads with low complexity regions and repeated sequences are identified and filtered by using the Komplexity (version 0.3.6) software (https://github.com/eclarke/komplexity) with default settings.

Metagenome assembly and annotation

Metagenome assembly is conducted by using MEGAHIT (version 1.0) [3] to assemble Qcpassed reads into contigs. As for taxonomy identification, two approaches are employed: (1) read-based taxonomy, which analyses QC-passed reads using Sourmash (version 2.0) [4] to estimate relative abundance of taxa based on GTDB taxonomy [5], and (2) contig-based taxonomy, which predicts taxonomy of contigs using MMSeqs2 [6, 7] easy-taxonomy pipeline and estimates abundance by contigs depth, which is derived by the jgi_summarize_bam_contig_depths script analysing bowtie2 alignment that maps QC-passed reads onto contigs. The open reading frame (ORF) prediction and functional annotation, including the Clusters of Orthologous Groups (COGs) family and Enzyme Commission (EC) number assignment, are performed by subjecting contigs into Prokka (version 1.14.5) [8]. ORFs are also analysed to identify corresponding KEGG Orthology (KO) numbers, Carbohydrate Active enZyme (CAZy) family, antibiotics gene, and virulence factor using MMSeqs2

easy-search pipeline. MinPath (version 1.1) [9] is used for pathway reconstruction by analysing KO (for KEGG pathway) and EC (for MetaCyc pathway [10]) profiles of each sample. The abundance of gene families (or categories) is estimated by accumulating ORF depths, which is calculated by the tpm_table python script (https://github.com/EnvGen/toolbox) based on the number of unique reads mapped on each ORF and presented in units of transcript per million (TPM) [11]. Metagenomics binning is performed by MetaBAT (version 2.17) [12] to cluster contigs into genome "bins" [i.e., metagenome-assembled genomes (MAGs)]. The quality is assessed, and the taxonomy is identified by CheckM (version 1.1.6) lineage-specific workflow [13]. Additionally, the taxonomy of MAGs is voted using the contig-based taxonomy of binned contigs, which extends the CheckM-predicted taxonomy to further depth in most cases.

Bioinformatics and statistical analysis

Microbial diversity was assessed using alpha diversity indices (Shannon, Simpson) and beta diversity using PERMANOVA with the "adonis2" function from the "vegan" package [18]. Principal Coordinate Analysis (PCoA) was used for ordination.

To evaluate associations between microbial taxa (metagenomic species, MGS) and metabolite concentrations, we performed linear regression models adjusted for age and sex. To account for multiple comparisons, **false discovery rate (FDR) correction was applied**, and both unadjusted and FDR-adjusted p-values are reported in the main manuscript (Table 2). Only FDR-significant associations were interpreted as robust.

In addition, we used LEfSe (linear discriminant analysis effect size) to identify differentially abundant taxa and gut metabolic modules between high and low metabolite groups. While LEfSe results are presented to aid visualization and exploratory discovery, interpretation was limited to associations validated through adjusted regression models. All plots were generated using "ggplot2" [17].

The initial step involved data preparation using the R package "phyloseq" [14], where samples were filtered, rare taxa removed, and species-level aggregation was performed. Patients were categorized into high and low metabolite groups using the median concentration for each metabolite. Group comparisons for clinical variables were conducted using t-tests, Wilcoxon rank-sum tests, or chi-square tests via the "compareGroups" package [15, 16]

Microbial diversity was assessed using alpha diversity indices (Shannon, Simpson) and beta diversity using PERMANOVA with the "adonis2" function from the "vegan" package [17]. Principal Coordinate Analysis (PCoA) was used for ordination. To evaluate associations between microbial taxa (metagenomic species, MGS) and metabolite concentrations, we performed linear regression models adjusted for age and sex. To account for multiple comparisons, false discovery rate (FDR) correction was applied, and both unadjusted and FDR-adjusted p-values are reported in the main manuscript (Table 2). Only FDR-significant associations were interpreted as robust. In addition, we used LEfSe (linear discriminant analysis effect size) to identify differentially abundant taxa and gut metabolic modules between high and low metabolite groups. While LEfSe results are presented to aid visualization and exploratory discovery, interpretation was limited to associations validated through adjusted regression models. All plots were generated using "ggplot2" [18]. Additionally, we also employed LEfSe analysis to identify differentially abundant features between high and low metabolite groups in the GMM pathway data [19, 20].

1. Bolger, A. M.; Lohse, M.; Usadel, B., Trimmomatic: a flexible trimmer for Illumina sequence data. *Bioinformatics* **2014,** 30, (15), 2114-2120.
2. Langmead, B.; Salzberg, S. L., Fast gapped-read alignment with Bowtie 2. *Nature methods* **2012,** 9, (4), 357-359.
3. Li, D.; Liu, C.-M.; Luo, R.; Sadakane, K.; Lam, T.-W., MEGAHIT: an ultra-fast single-node solution for large and complex metagenomics assembly via succinct de Bruijn graph. *Bioinformatics* **2015,** 31, (10), 1674-1676.
4. Pierce, N. T.; Irber, L.; Reiter, T.; Brooks, P.; Brown, C. T., Large-scale sequence comparisons with sourmash. *F1000Research* **2019,** 8.
5. Parks, D. H.; Chuvochina, M.; Waite, D. W.; Rinke, C.; Skarshewski, A.; Chaumeil, P.-A.; Hugenholtz, P., A standardized bacterial taxonomy based on genome phylogeny substantially revises the tree of life. *Nature biotechnology* **2018,** 36, (10), 996-1004.
6. Mirdita, M.; Steinegger, M.; Breitwieser, F.; Söding, J.; Levy Karin, E., Fast and sensitive taxonomic assignment to metagenomic contigs. *Bioinformatics* **2021,** 37, (18), 3029-3031.
7. Steinegger, M.; Söding, J., MMseqs2 enables sensitive protein sequence searching for the analysis of massive data sets. *Nature biotechnology* **2017,** 35, (11), 1026-1028.
8. Seemann, T., Prokka: rapid prokaryotic genome annotation. *Bioinformatics* **2014,** 30, (14), 2068-2069.
9. Ye, Y.; Doak, T. G., A parsimony approach to biological pathway

reconstruction/inference for genomes and metagenomes. *PLoS computational biology* **2009,** 5, (8), e1000465.
10. Caspi, R.; Billington, R.; Keseler, I. M.; Kothari, A.; Krummenacker, M.; Midford, P. E.; Ong, W. K.; Paley, S.; Subhraveti, P.; Karp, P. D., The MetaCyc database of metabolic pathways and enzymes-a 2019 update. *Nucleic acids research* **2020,** 48, (D1), D445-D453.
11. Wagner, G. P.; Kin, K.; Lynch, V. J., Measurement of mRNA abundance using RNA-seq data: RPKM measure is inconsistent among samples. *Theory in biosciences* **2012,** 131, 281-285.
12. Kang, D. D.; Li, F.; Kirton, E.; Thomas, A.; Egan, R.; An, H.; Wang, Z., MetaBAT 2: an adaptive binning algorithm for robust and efficient genome reconstruction from metagenome assemblies. *PeerJ* **2019,** 7, e7359.
13. Parks, D. H.; Imelfort, M.; Skennerton, C. T.; Hugenholtz, P.; Tyson, G. W., CheckM: assessing the quality of microbial genomes recovered from isolates, single cells, and metagenomes. *Genome research* **2015,** 25, (7), 1043-1055.
14. McMurdie, P. J.; Holmes, S., phyloseq: an R package for reproducible interactive analysis and graphics of microbiome census data. *PloS one* **2013,** 8, (4), e61217.
15. Alonso A, M. S. compareGroups: a free R package for descriptive analysis.
16. Alonso A, M. S., Descriptive Analysis by Groups. R package version 4.6.0. 2022. In 2022.
17. Oksanen J, B. F., Friendly M, et al., vegan: Community Ecology Package. R package version 2.6-4. In 2022.
18. Wickham, H.; Wickham, H., Data analysis. *ggplot2: elegant graphics for data analysis* **2016**, 189-201.
19. Segata, N.; Izard, J.; Waldron, L.; Gevers, D.; Miropolsky, L.; Garrett, W. S.; Huttenhower, C., Metagenomic biomarker discovery and explanation. *Genome biology* **2011,** 12, 1-18.
20. Cao, Y.; Dong, Q.; Wang, D.; Zhang, P.; Liu, Y.; Niu, C., microbiomeMarker: an R/Bioconductor package for microbiome marker identification and visualization. *Bioinformatics* **2022,** 38, (16), 4027-4029.

Supplementary Figures

Identification of gut microbiome signatures associated with serotonin pathway in tryptophan metabolism in patients undergoing hemodialysis

Supplementary Figure S1. Genus- and species-level relative abundances stratified by high vs. low 5-HTP levels.

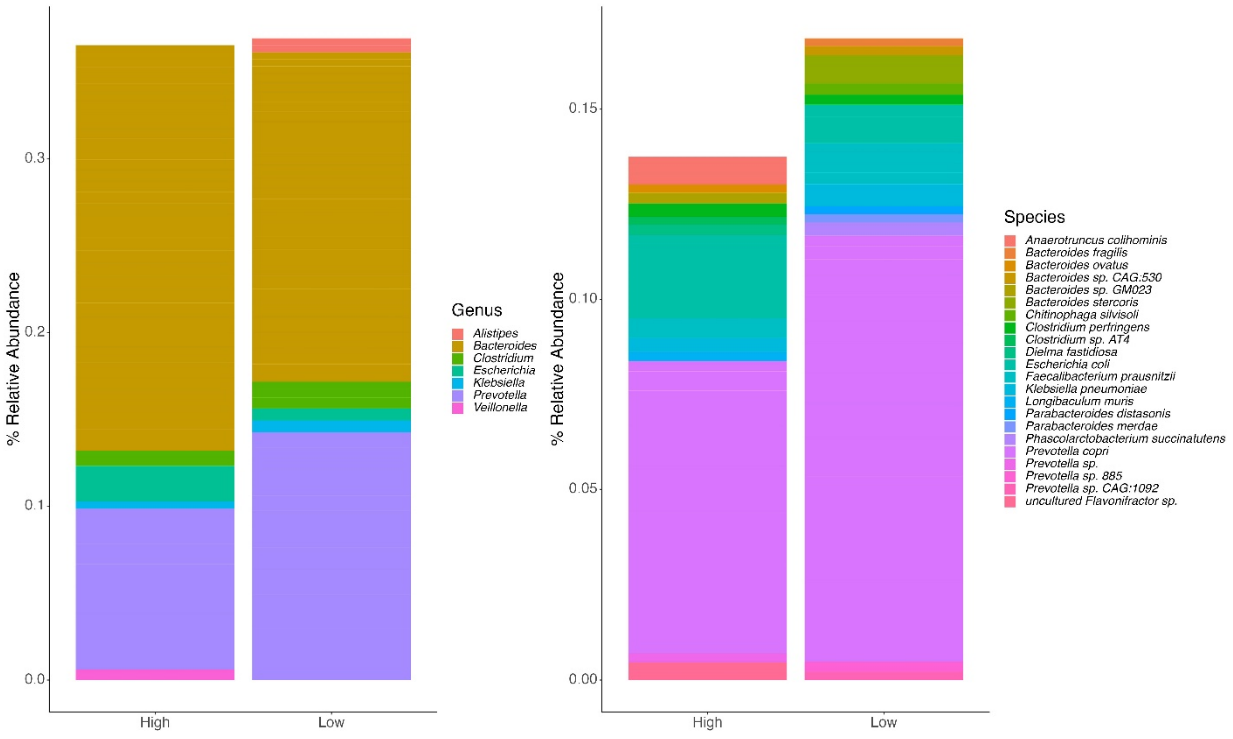

Supplementary Figure S2. Genus- and species-level relative abundances stratified by high vs. low serotonin levels.

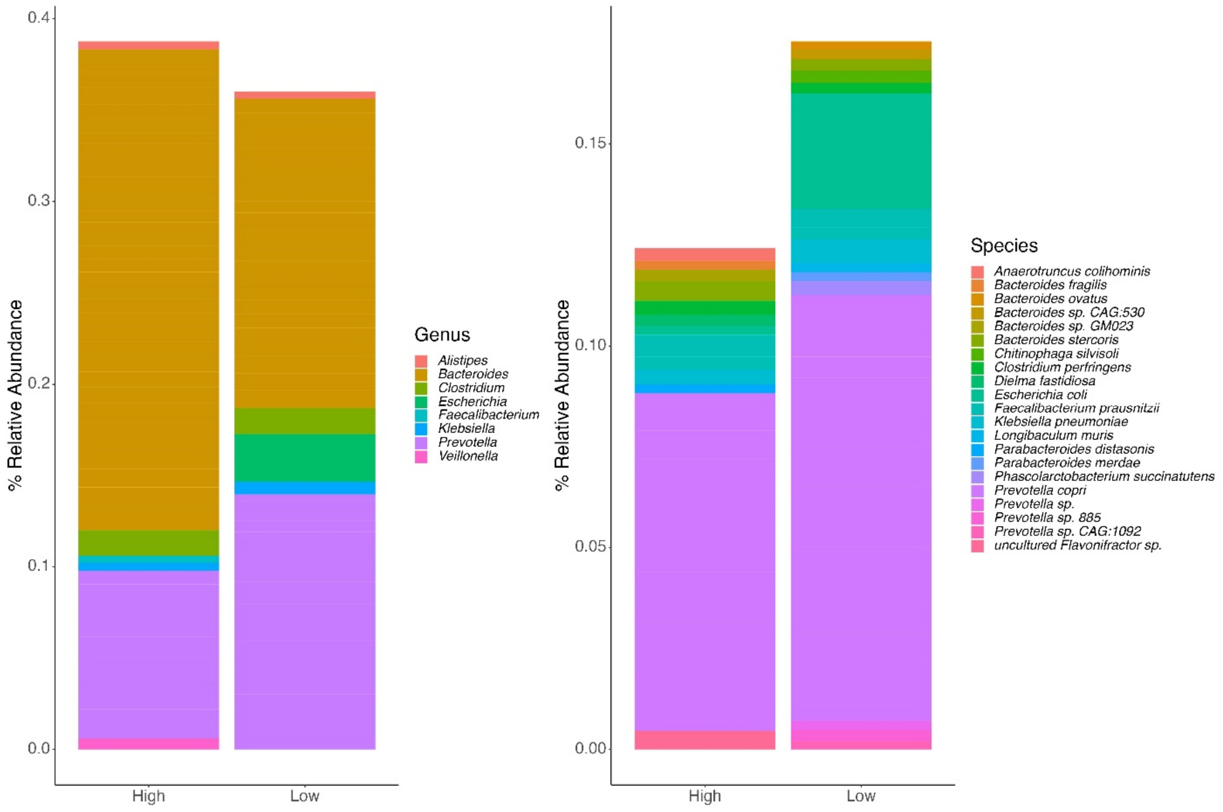

Supplementary Figure S3. Genus- and species-level relative abundances stratified by high vs. low 5-MTP levels.

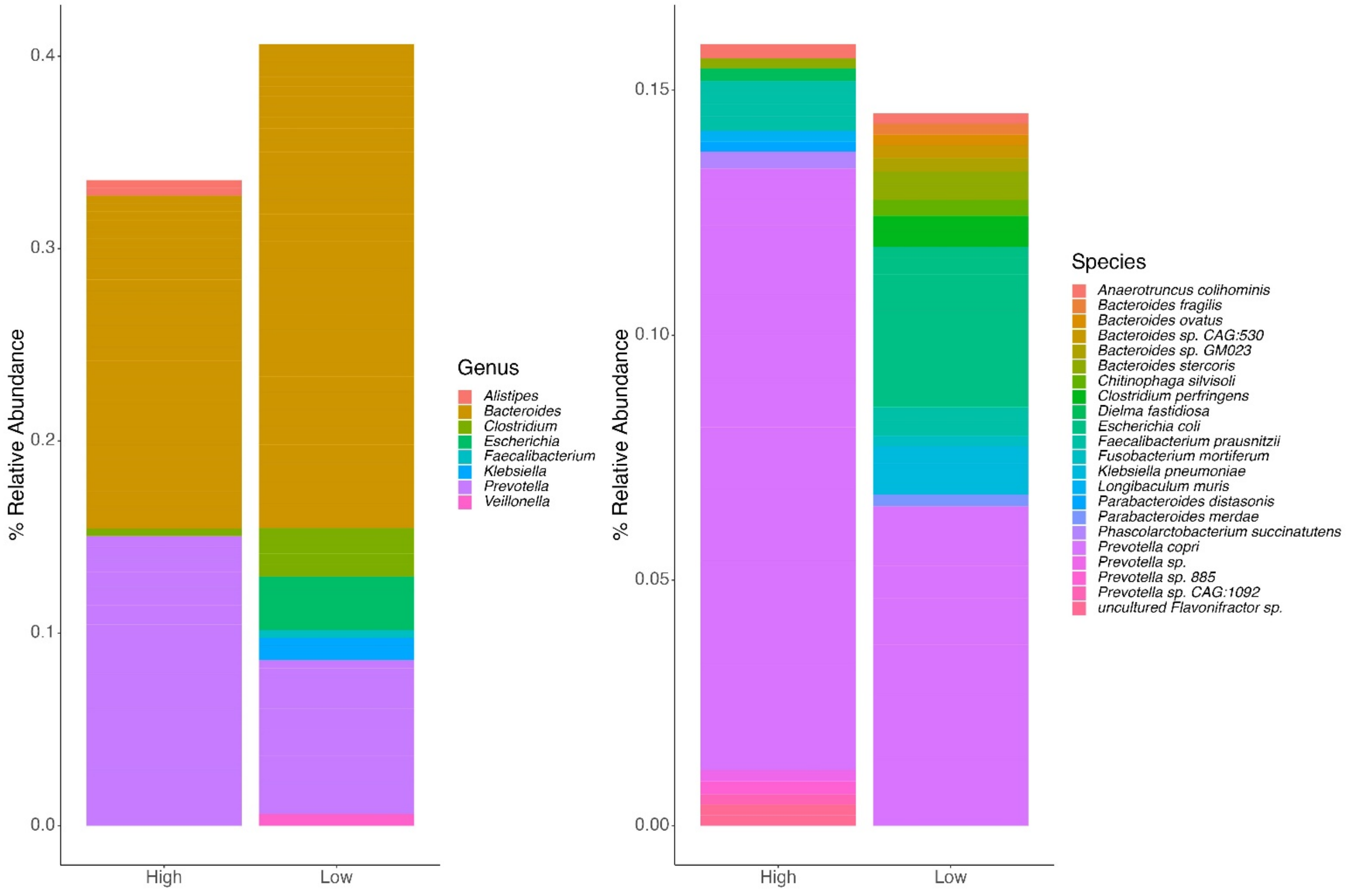

Supplementary Figure S4. Genus- and species-level relative abundances stratified by high vs. low 5-methoxytryptamine levels.

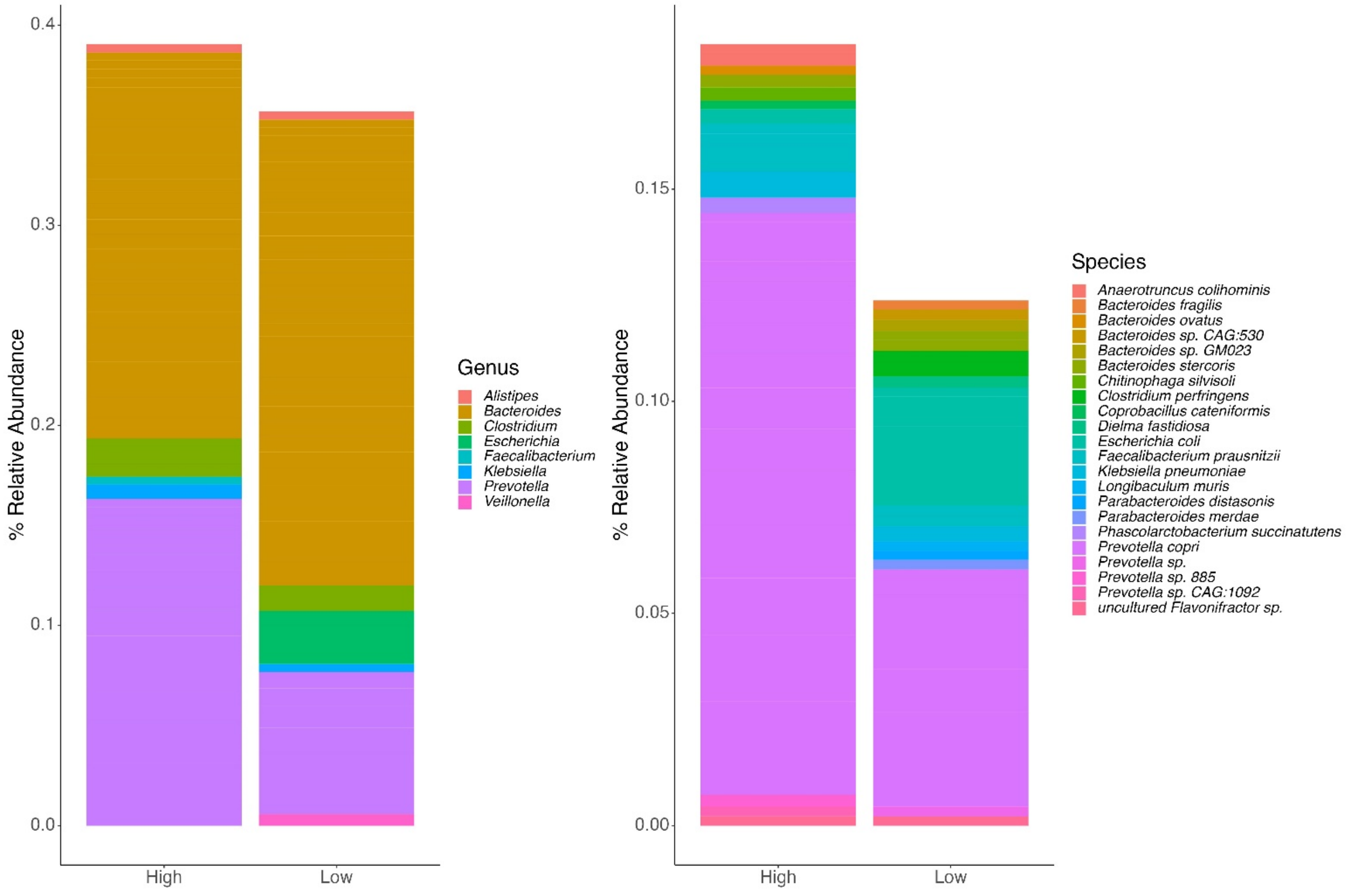

Supplementary Figure S5. Genus- and species-level relative abundances stratified by high vs. low melatonin levels.

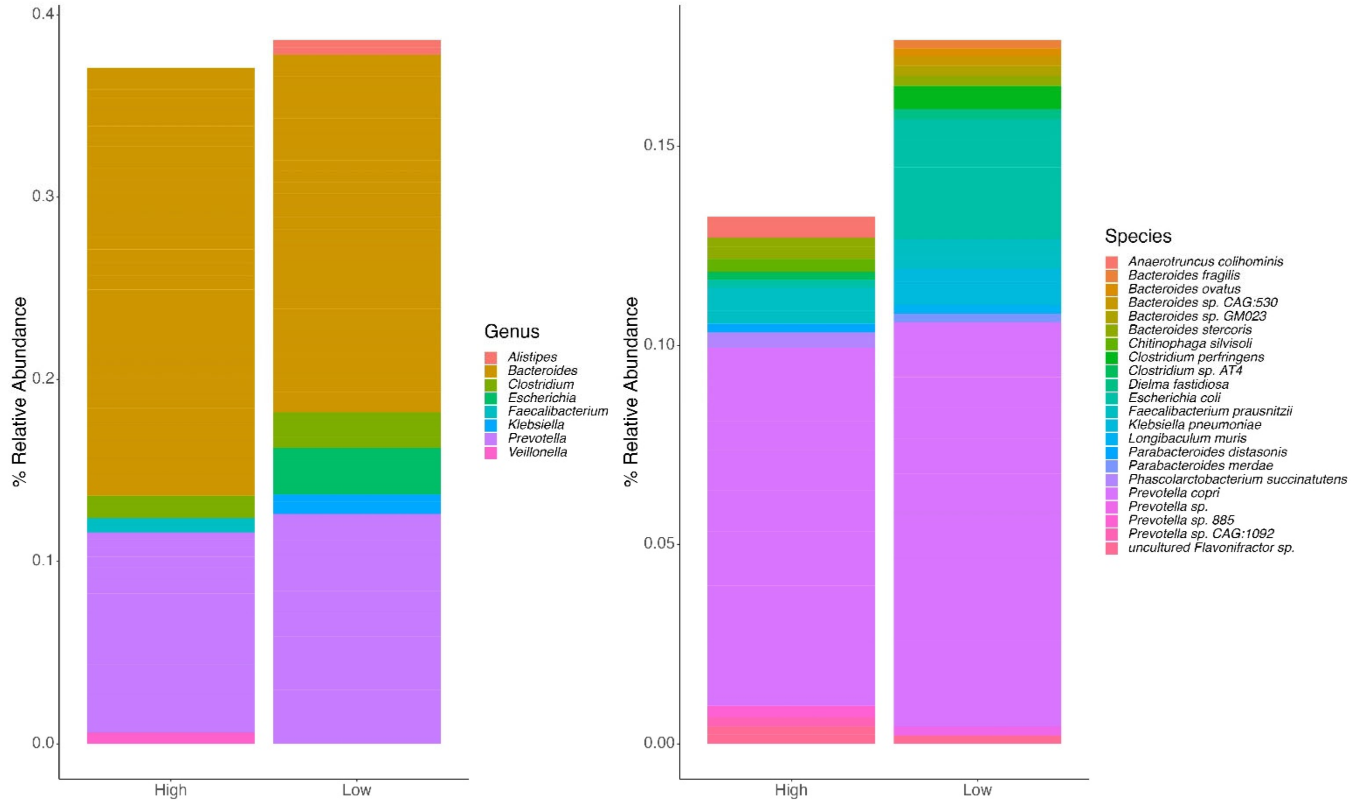

Supplementary Figure S6. Genus- and species-level relative abundances stratified by high vs. low 6-hydroxymelatonin levels.

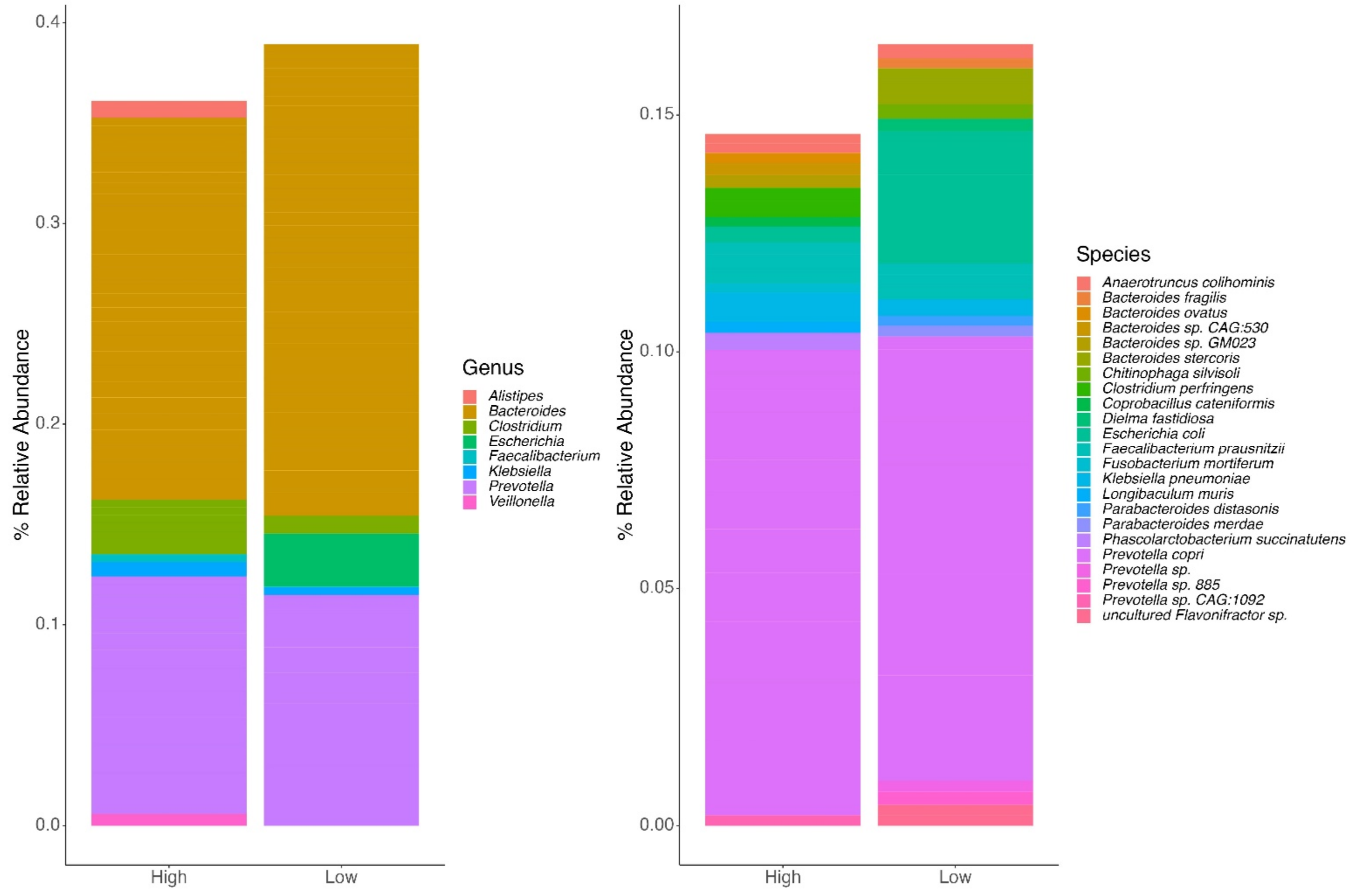

Supplementary Figure S7. LEfSe (Linear Discriminant Analysis Effect Size) cladogram showing taxa differentially abundant between high and low serotonin groups. Nodes are colored according to the group in which they are enriched. The taxonomic prefixes indicate rank: *s_* = species, *g_* = genus, *f_* = family, *o_* = order, *c_* = class.

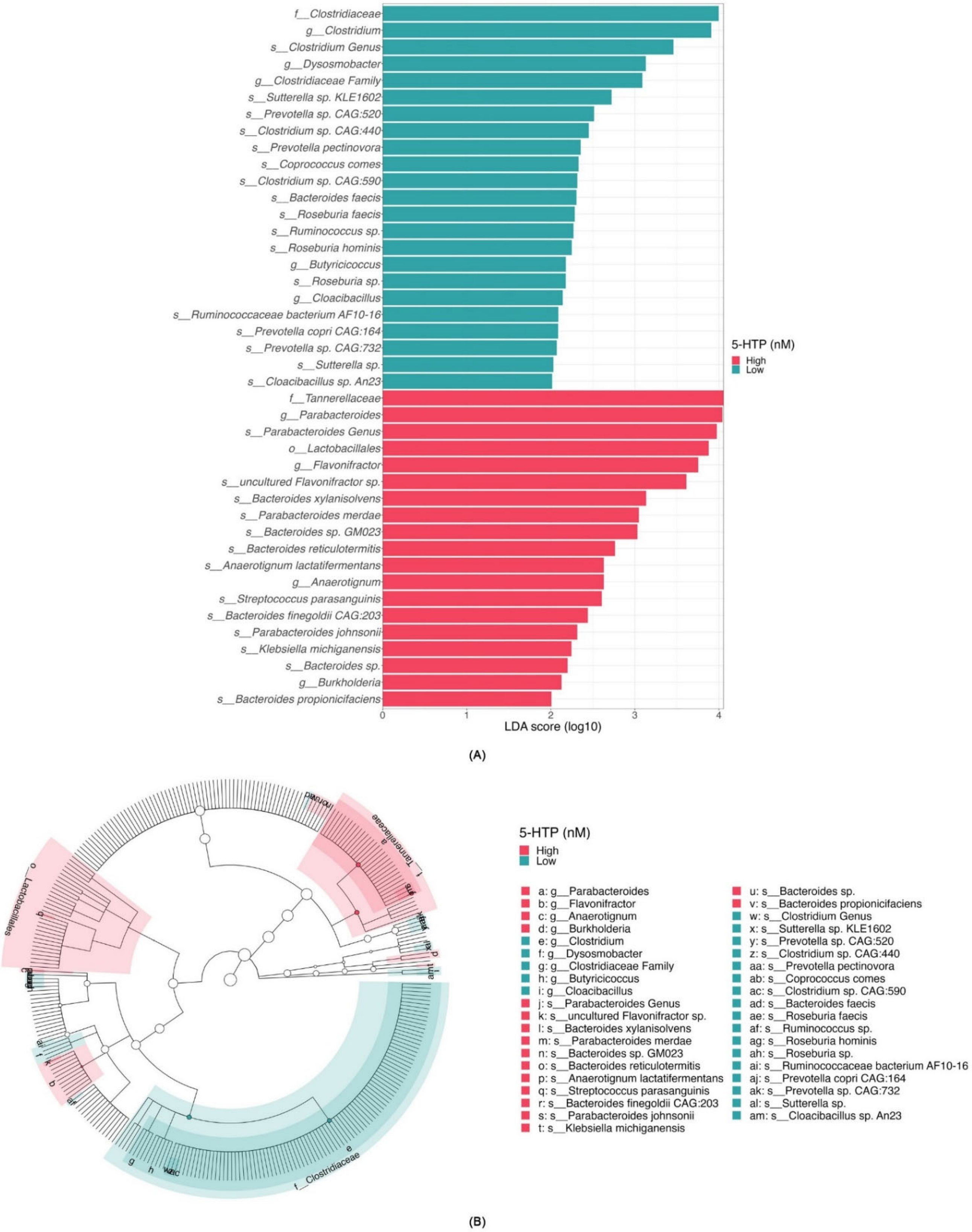

Supplementary Figure S8. LEfSe cladogram showing taxa differentially abundant between high and low 5-HTP groups. Prefixes denote taxonomic rank (*s_* = species, *g_* = genus, *f_* = family, *o_* = order, *c_* = class). Colored circles highlight taxa enriched in each group, with circle size reflecting relative abundance.

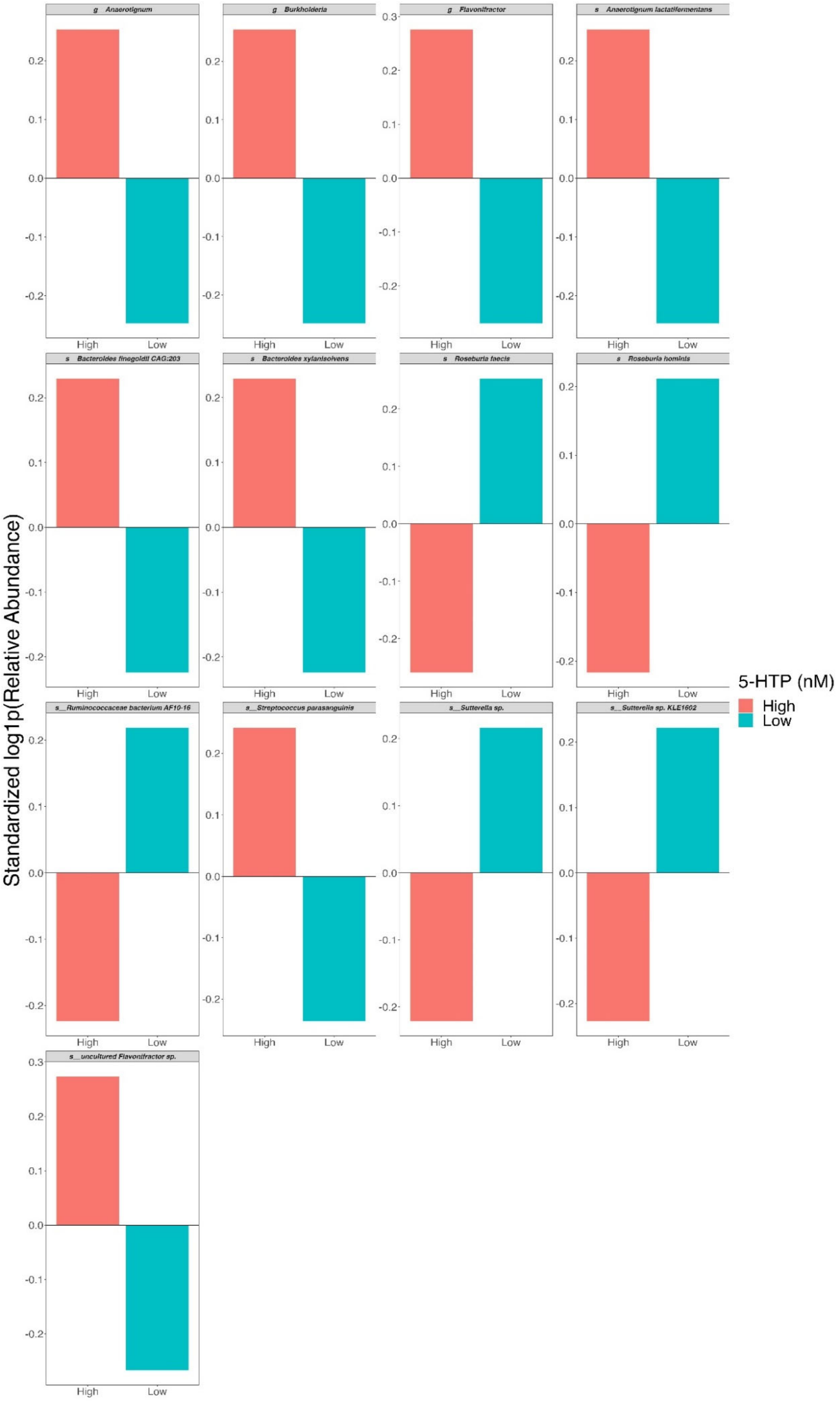

Supplementary Figure S9. LEfSe (Linear Discriminant Analysis Effect Size) Analysis of Gut Metabolic Modules Based on the Level of 5-HTP.

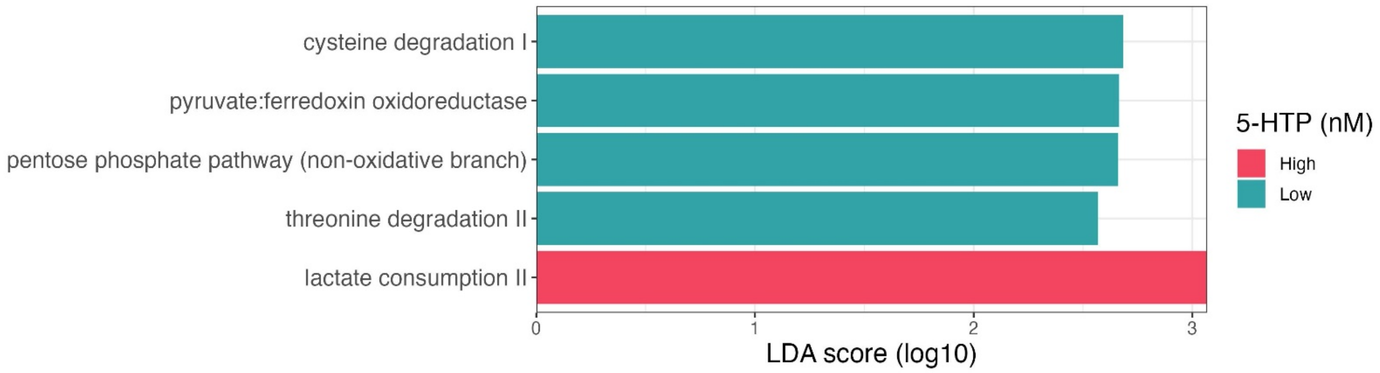

Supplementary Figure S10. LEfSe (Linear Discriminant Analysis Effect Size) Analysis and Cladogram Construction for the Microbiome Associated with Serotonin.

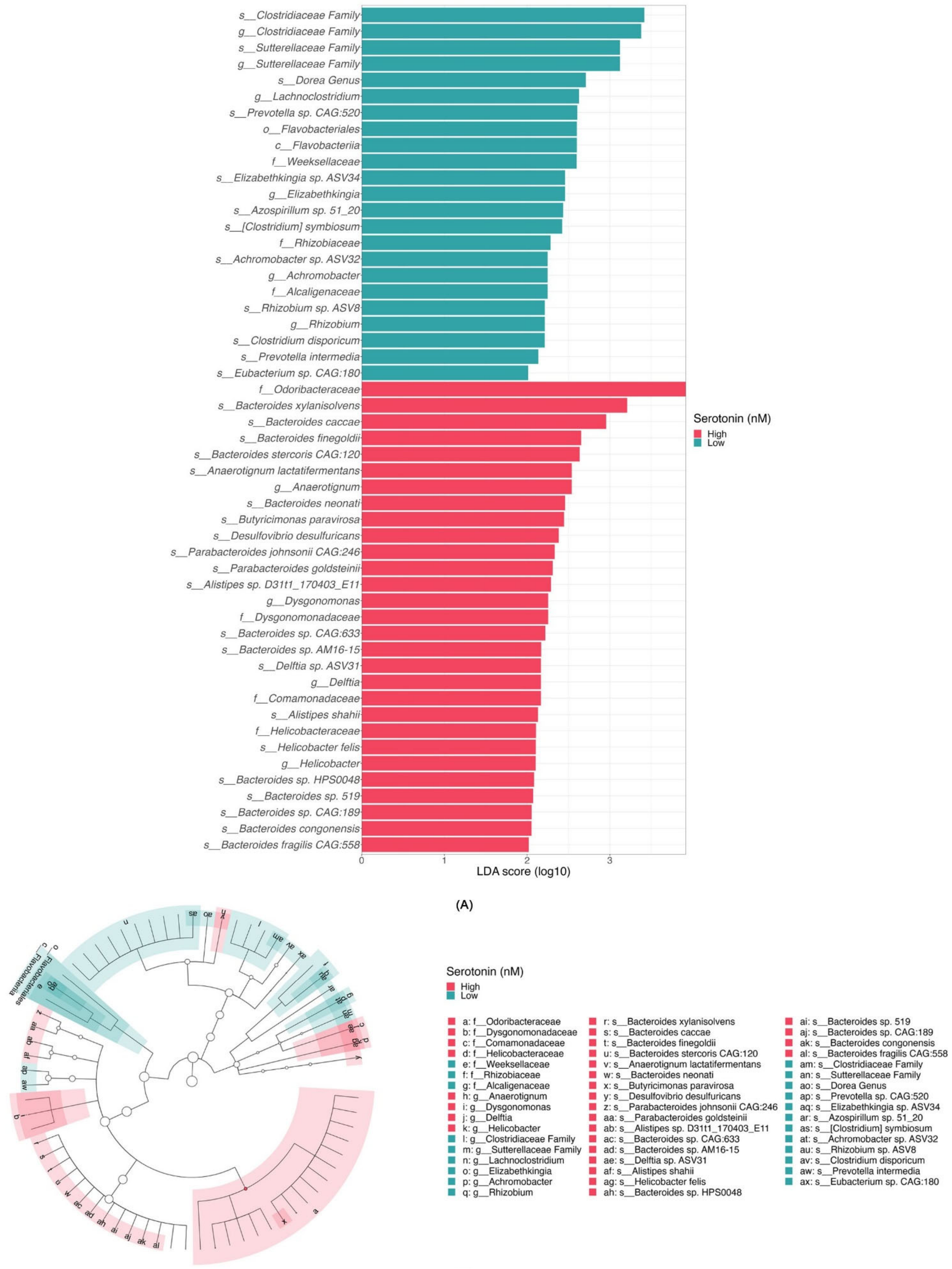

(B)

Supplementary Figure S11. The Comparison of Standardized Relative Abundance Between High and Low Levels of Serotonin.

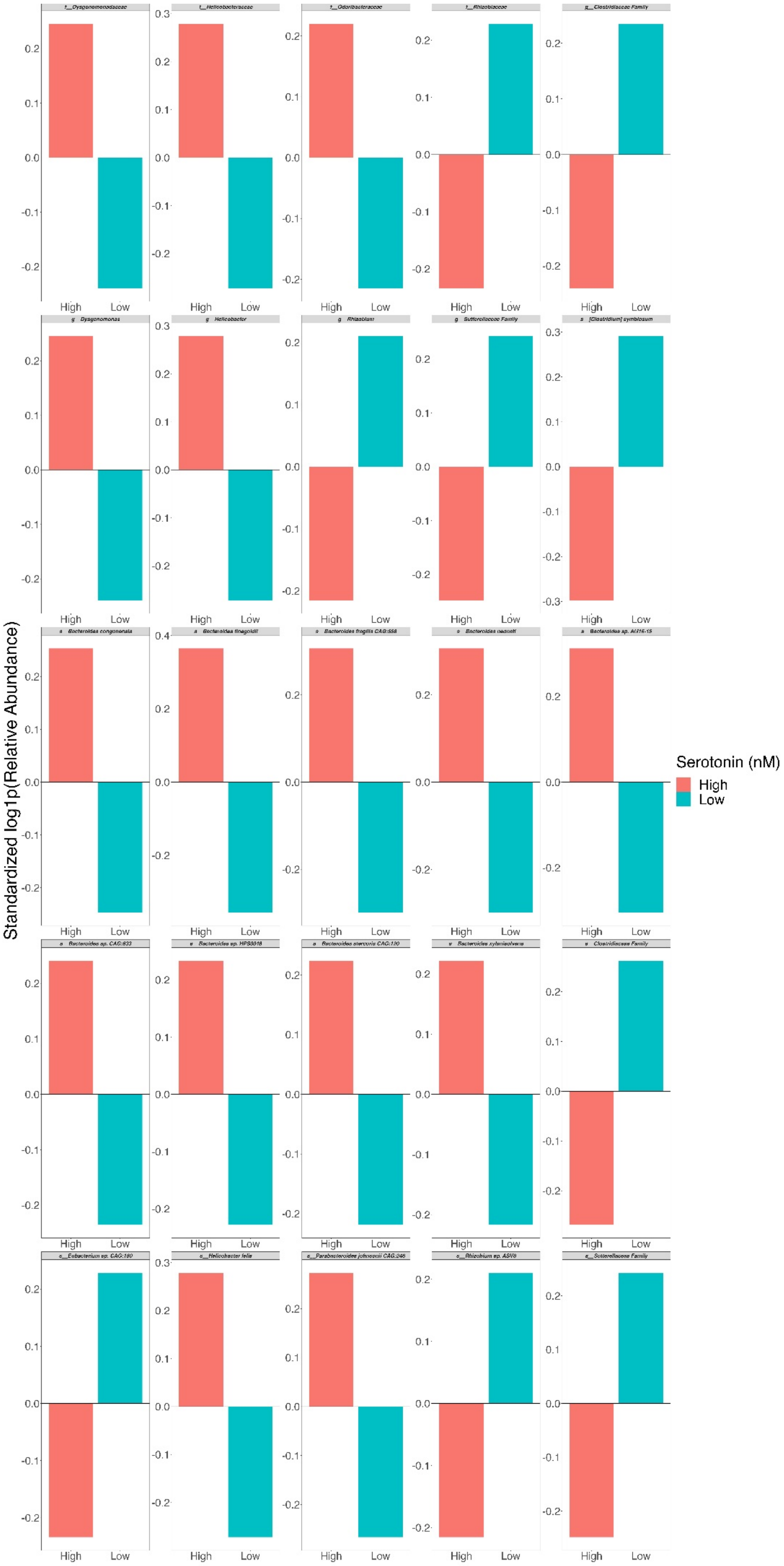

Supplementary Figure S12. LEfSe (Linear Discriminant Analysis Effect Size) Analysis of Gut Metabolic Modules Based on the Level of Serotonin.

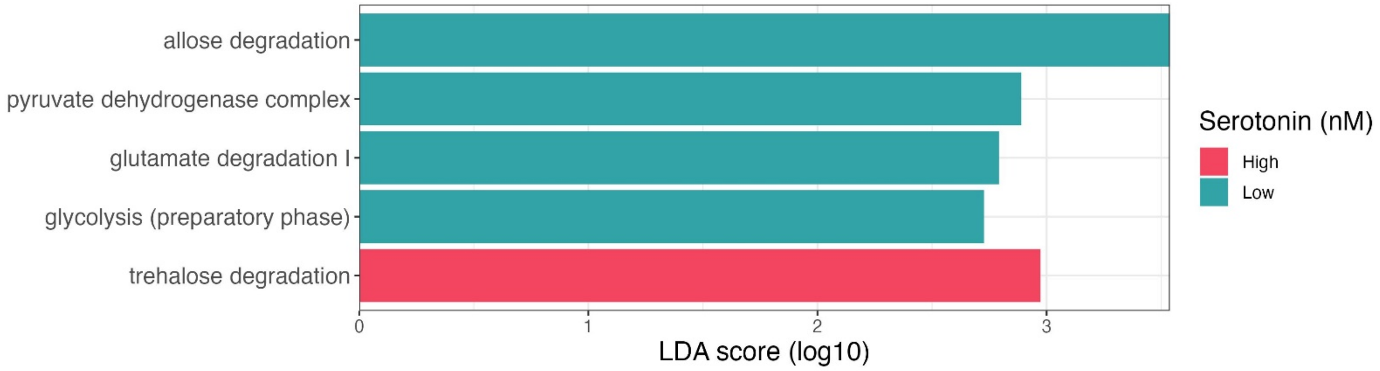

Supplementary Figure S13. LEfSe cladogram showing taxa differentially abundant between high and low 5-MTP groups. Taxonomic prefixes: *s_* = species, *g_* = genus, *f_* = family, *o_* = order, *c_* = class.

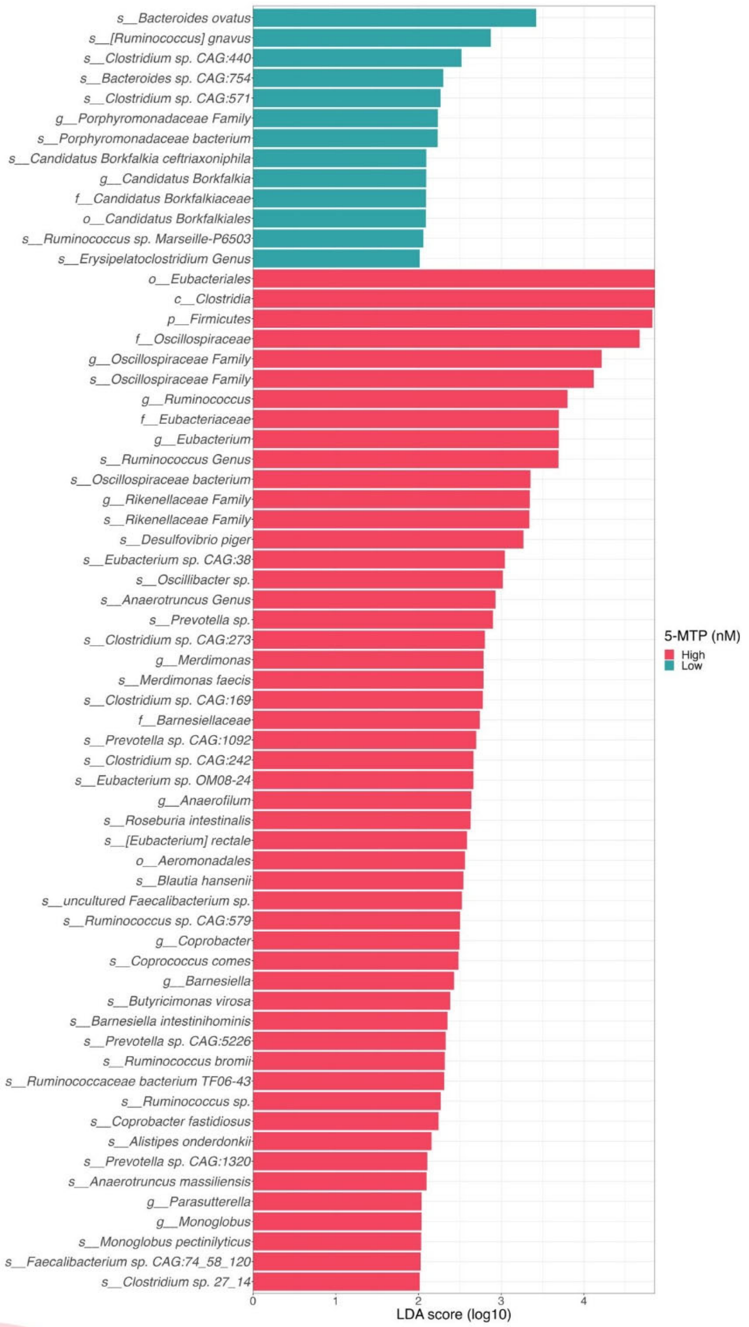

(A)

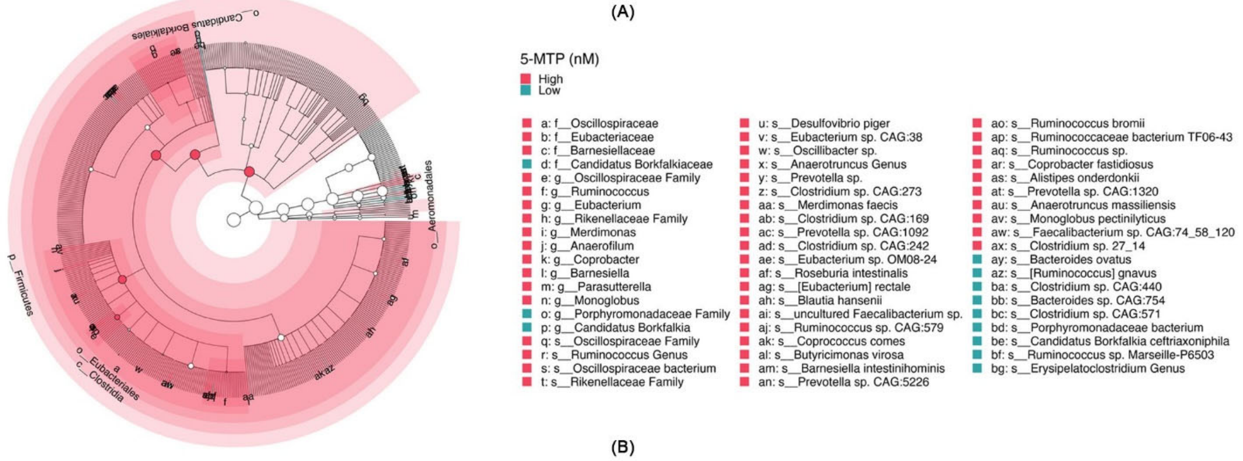

(B)

Supplementary Figure S14. The Comparison of Standardized Relative Abundance Between High and Low Levels of 5-MTP.

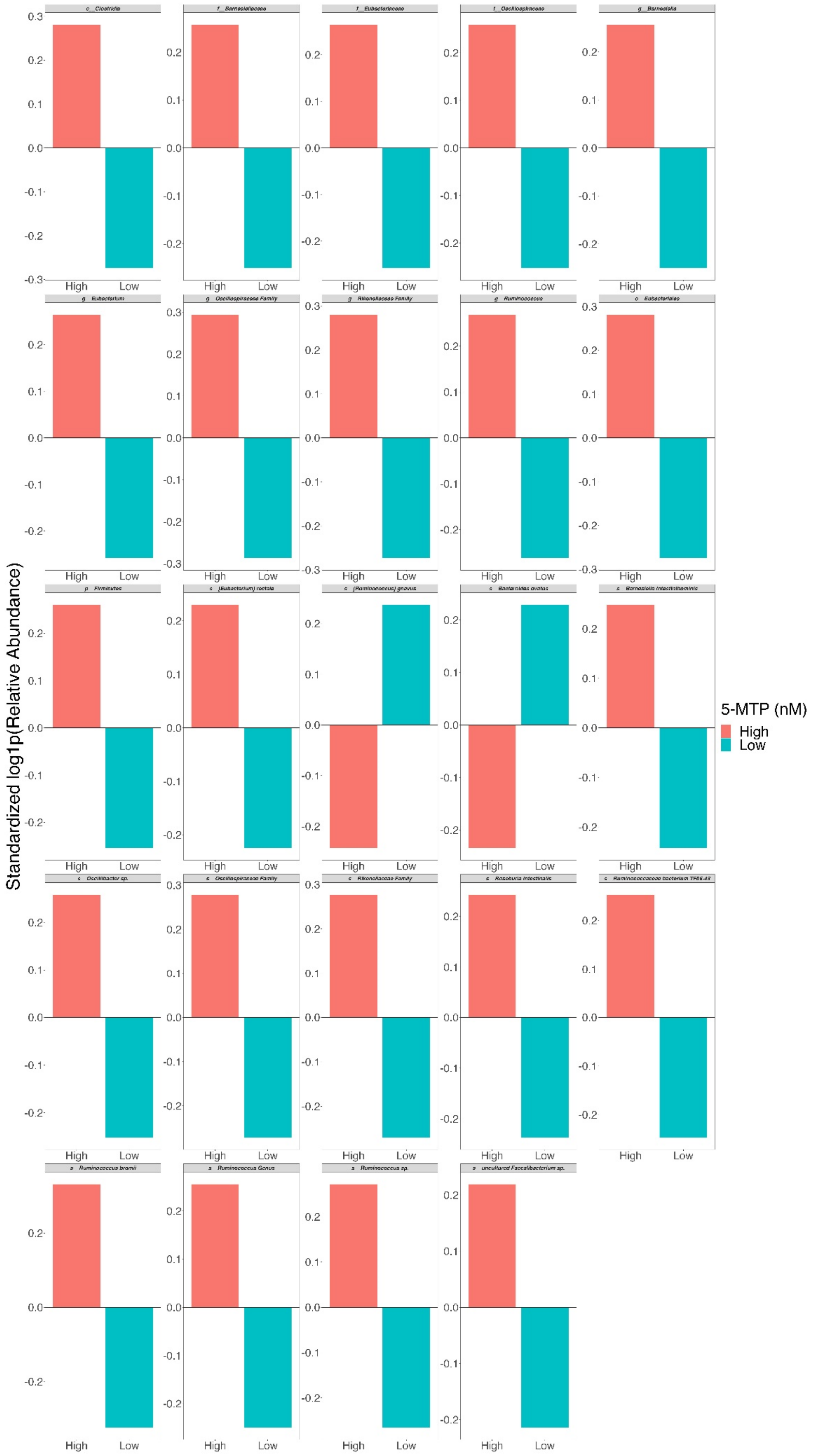

Supplementary Figure S15. LEfSe (Linear Discriminant Analysis Effect Size) Analysis of Gut Metabolic Modules Based on the Level of 5-MTP.

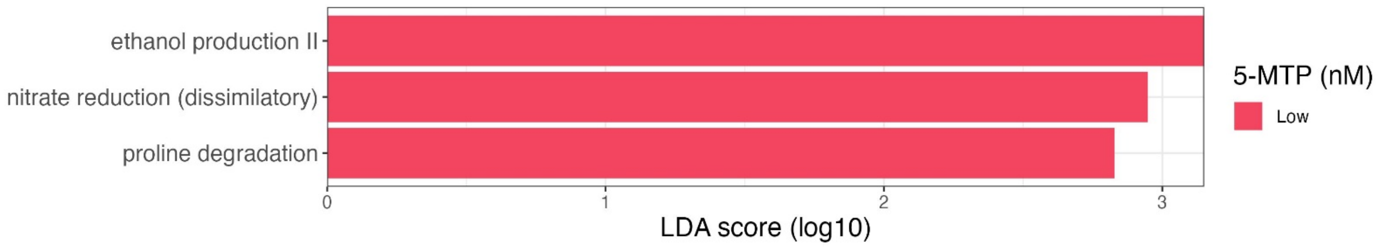

Supplementary Figure S16. LEfSe cladogram showing taxa differentially abundant between high and low 5-methoxytryptamine groups. Prefixes indicate taxonomic rank (*s_* = species, *g_* = genus, *f_* = family, *o_* = order, *c_* = class).

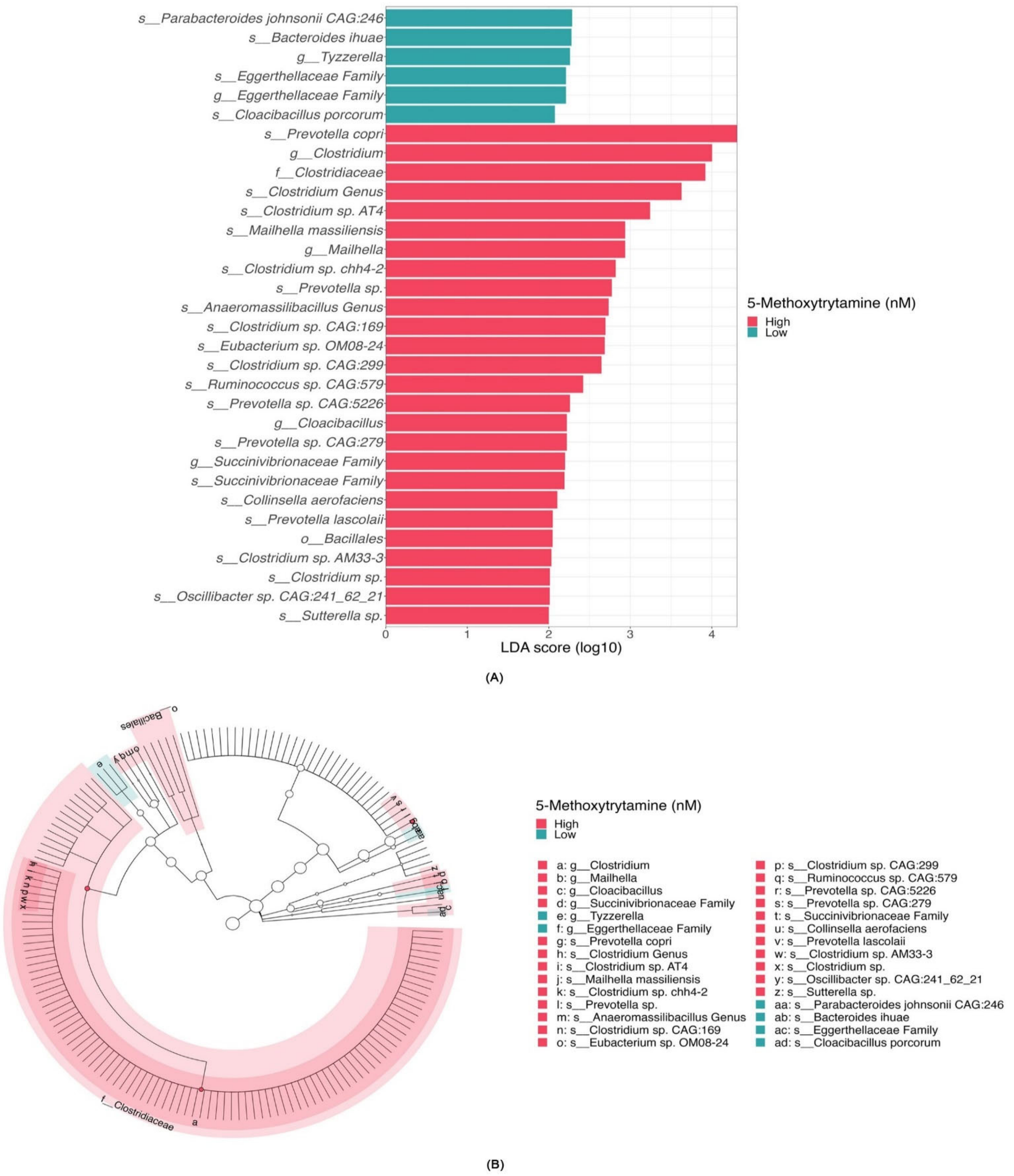

Supplementary Figure S17. The Comparison of Standardized Relative Abundance Between High and Low Levels of 5-Methoxytryptamine.

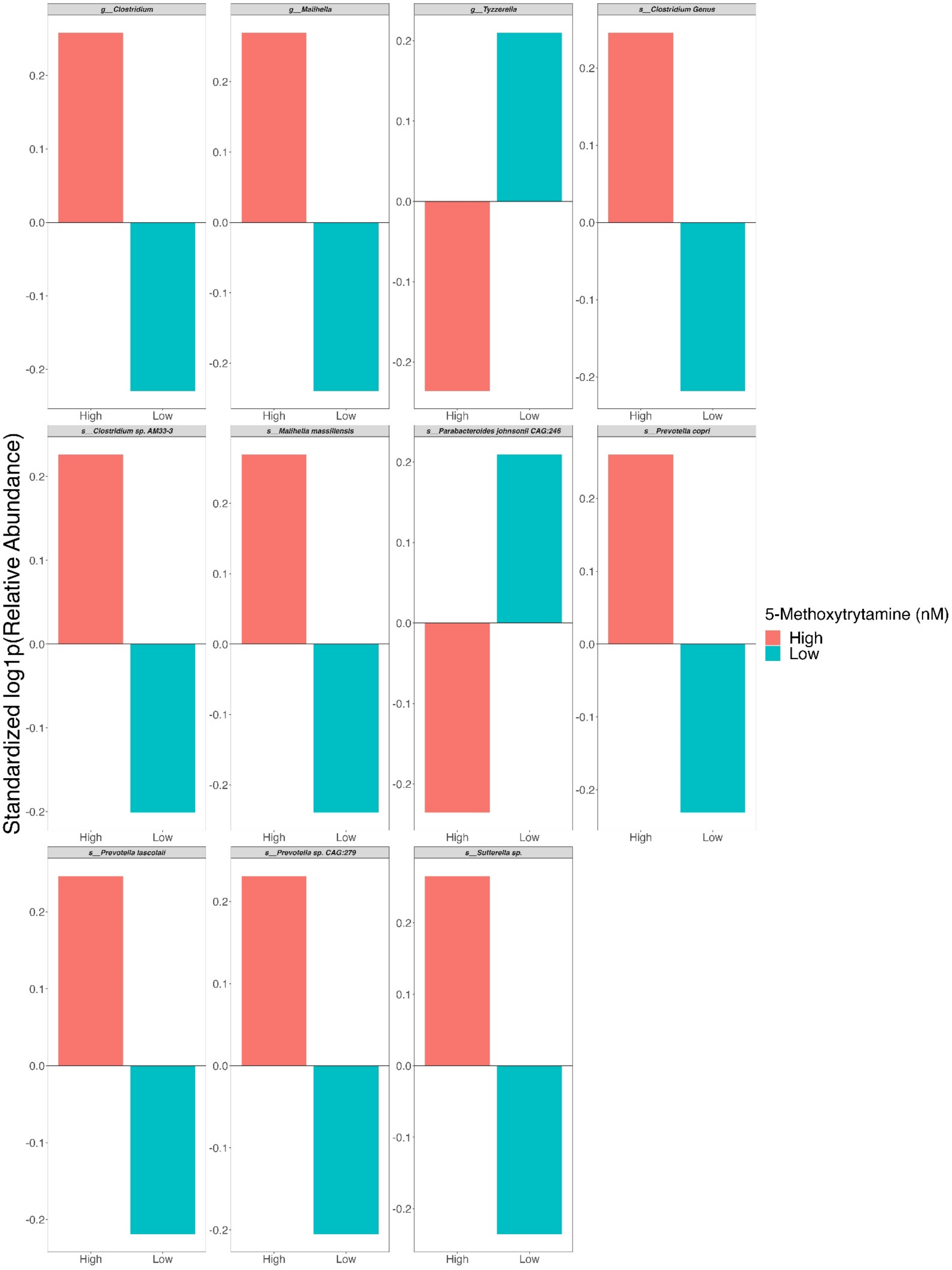

Supplementary Figure S18. LEfSe (Linear Discriminant Analysis Effect Size) Analysis of Gut Metabolic Modules Based on the Level of 5-Methoxytryptamine.

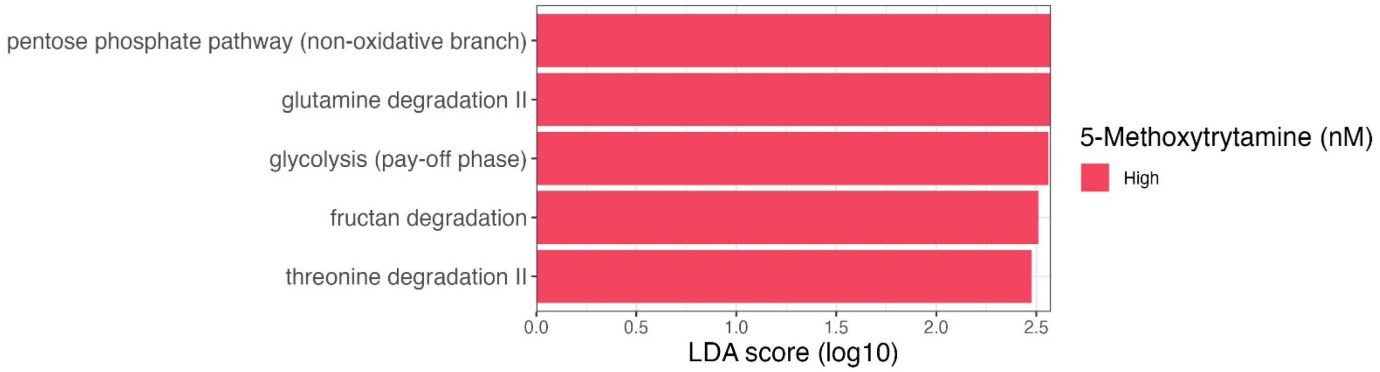

Supplementary Figure S19. LEfSe cladogram showing taxa differentially abundant between high and low melatonin groups. Taxonomic prefixes are as follows: *s_* = species, *g_* = genus, *f_* = family, *o_* = order, *c_* = class.

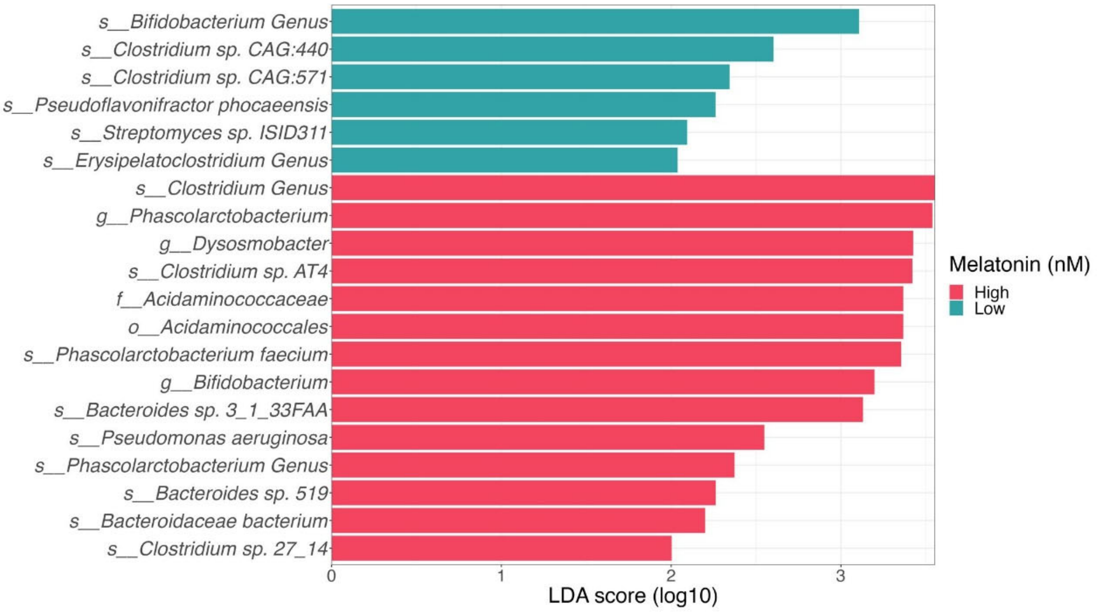

(A)

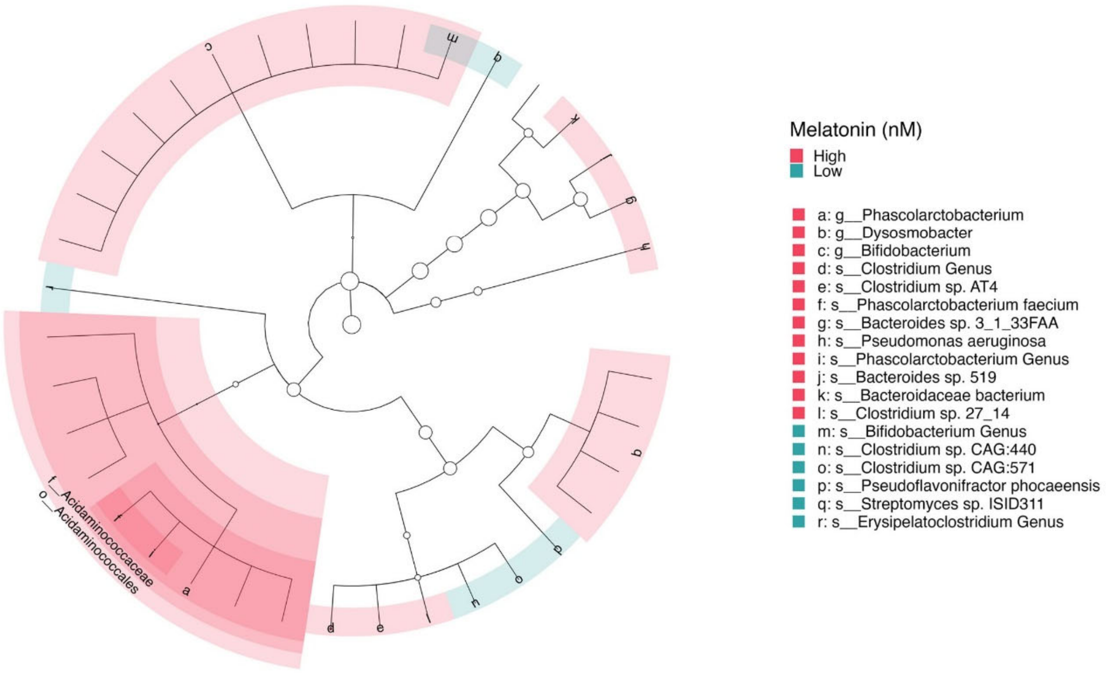

(B)

Supplementary Figure S20. The Comparison of Standardized Relative Abundance Between High and Low Levels of Melatonin

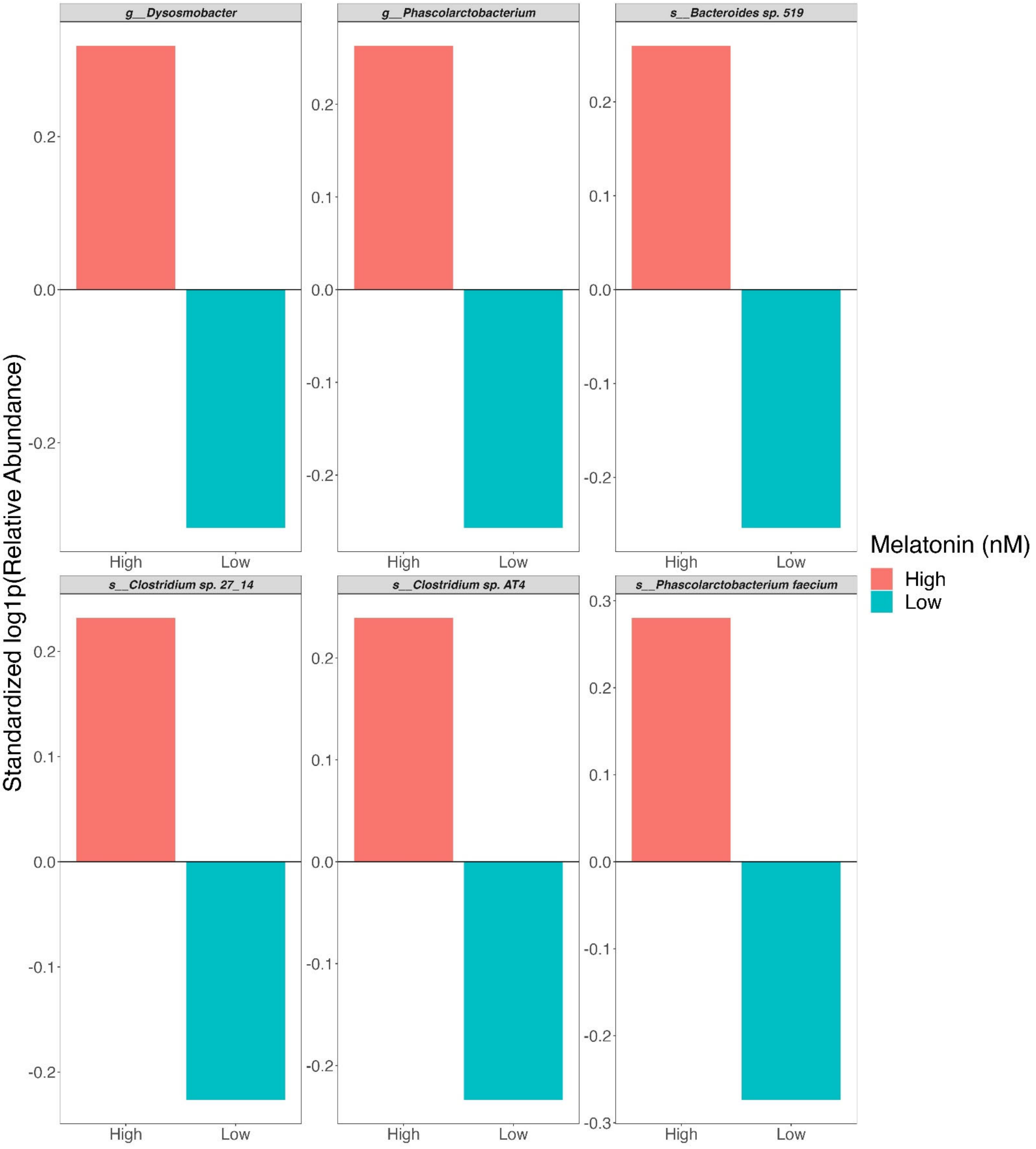

Supplementary Figure S21. LEfSe (Linear Discriminant Analysis Effect Size) Analysis of Gut Metabolic Modules Based on the Level of Melatonin.

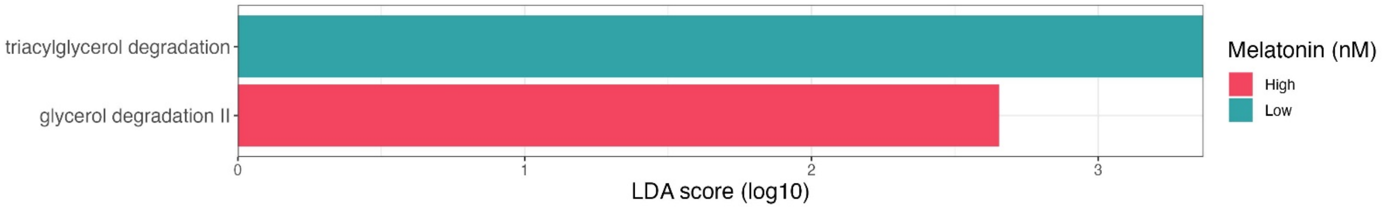

Supplementary Figure S22. LEfSe cladogram showing taxa differentially abundant between high and low 6-hydroxymelatonin groups. Prefixes: *s_* = species, *g_* = genus, *f_* = family, *o_* = order, *c_* = class.

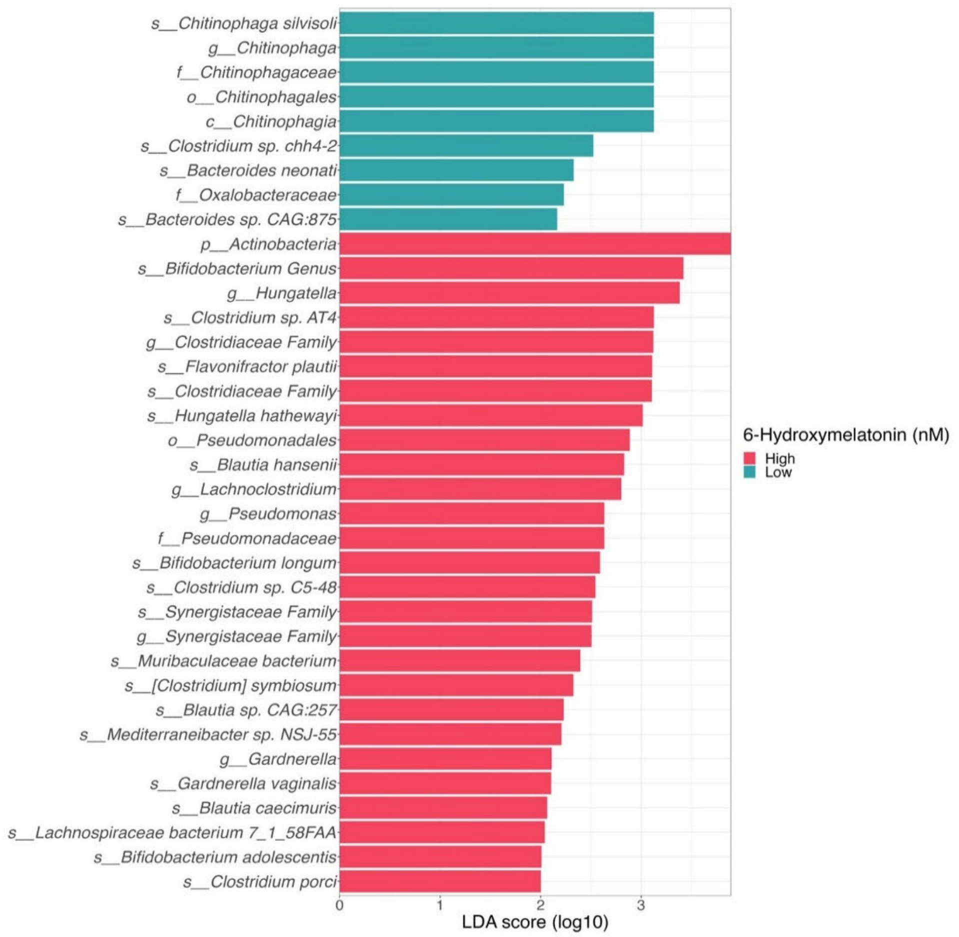

(A)

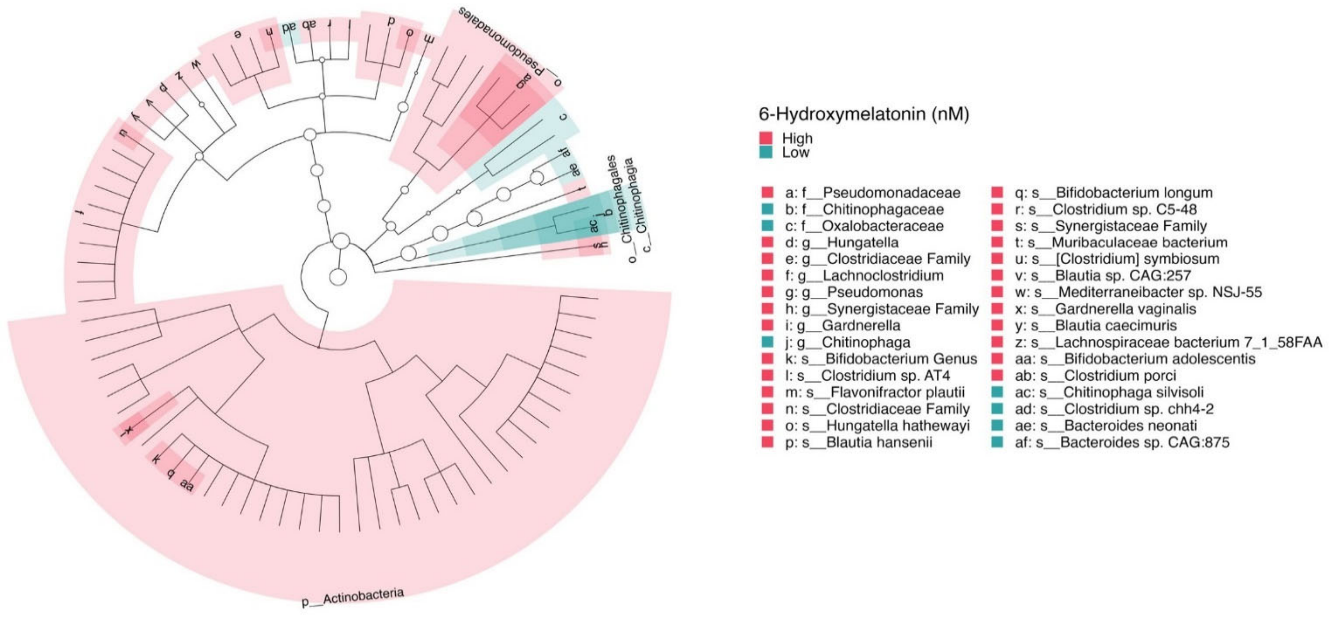

(B)

Supplementary Figure S23. The Comparison of Standardized Relative Abundance Between High and Low Levels of 6-Hydroxymelatonin

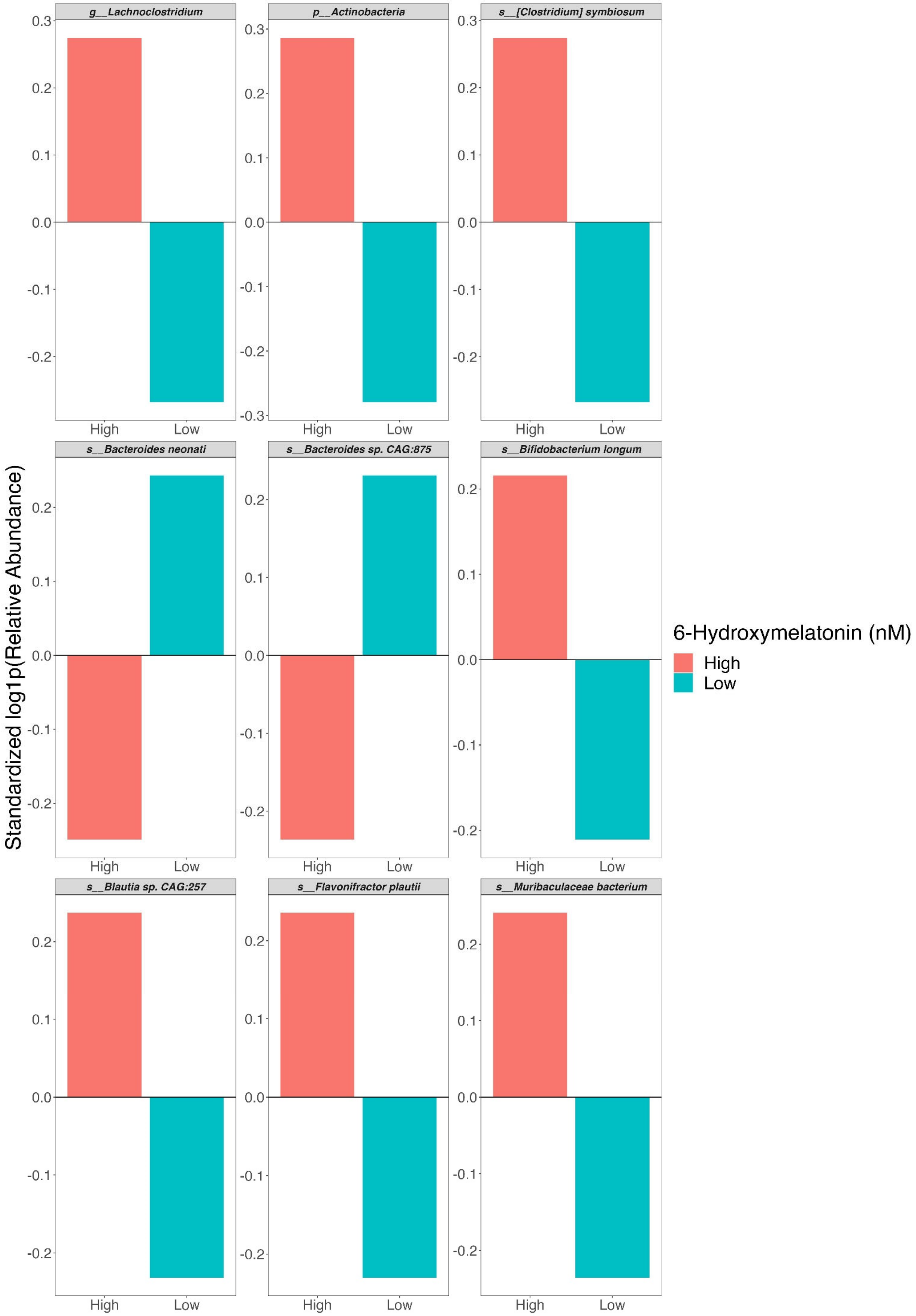

Supplementary Figure S24. LEfSe (Linear Discriminant Analysis Effect Size) Analysis of Gut Metabolic Modules Based on the Level of 6-Hydroxymelatonin.

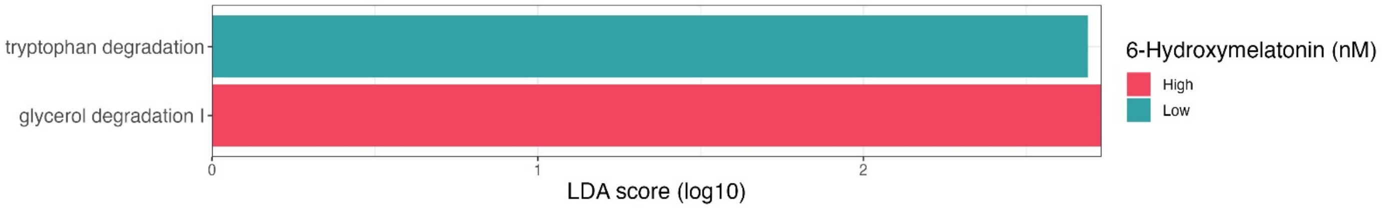

Supplement: Supplementary file 1 [file ijms-26-10463-s001.zip › ijms-3834836-supplementary.pdf]
